# TDP-43 overexpression in the hypothalamus drives neuropathology, dysregulates metabolism and impairs behavior in mice

**DOI:** 10.1186/s40478-025-02018-8

**Published:** 2025-05-27

**Authors:** Sofia Bergh, Nicolas Casadei, Sanaz Gabery, Oskar Simonsson, João M. N. Duarte, Deniz Kirik, Huu Phuc Nguyen, Åsa Petersén

**Affiliations:** 1 Translational Neuroendocrine Research Unit (TNU), Department of Experimental Medical Science, Lund University, BMC D11, Lund, 221 84 Sweden; 2 Institute of Medical Genetics and Applied Genomics, University of Tübingen, Tübingen, Germany; 3 NGS Competence Center Tübingen, Tübingen, Germany; 4 Diabetes and Brain Function Unit, Department of Experimental Medical Science, Lund University, Lund, Sweden; 5 Wallenberg Centre for Molecular Medicine, Lund University, Lund, Sweden; 6 Brain Repair and Imaging in Neural Systems (BRAINS), Department of Experimental Medical Science, Lund University, Lund, Sweden; 7 Department of Human Genetics, Medical Faculty, Ruhr University Bochum, Bochum, Germany; 8 Department of Psychiatry, Skåne University Hospital, Lund, Sweden

**Keywords:** (4 to 6): TDP-43, Hypothalamus, Metabolism, Amyotrophic lateral sclerosis, Frontotemporal dementia

## Abstract

TAR DNA-binding protein 43 (TDP-43) pathology is linked to the neurodegenerative disorders amyotrophic lateral sclerosis (ALS), frontotemporal dementia (FTD) and Huntington disease (HD). Dysregulation of metabolism and emotion is shared across these disorders and may be caused by hypothalamic pathology. Inclusions with TDP-43 are present in the hypothalamus in clinical ALS, as well as selective loss of hypothalamic neurons expressing the metabolism and emotion regulating neuropeptides hypocretin (orexin), melanin-concentrating hormone (MCH) and oxytocin. We aimed to investigate whether there is a casual link between the effects of TDP-43 in the hypothalamus and the development of neuropathology, as well as changes in metabolism and behavior. We generated an adeno-associated viral (AAV) vector expressing human TDP-43 under the neuronal-specific synapsin promoter, which was injected bilaterally into the hypothalamus of wild-type FVB/N mice. TDP-43 overexpression resulted in hypothalamic pathology in a dose-dependent fashion replicating clinical pathology with hypothalamic atrophy and loss of hypocretin-, MCH- and oxytocin-expressing neurons. Nuclear and cytoplasmic inclusions of TDP-43 were found in the hypothalamus. Mice overexpressing TDP-43 in the hypothalamus developed metabolic dysregulation with hyperglycaemia independent of food intake. Additionally, mice overexpressing TDP-43 in the hypothalamus exhibited reduced motor activity and nesting ability, suggesting the development of an apathy-like phenotype. Taken together, AAV-vector mediated TDP-43 overexpression in the hypothalamus leads to neuropathology with the development of metabolic dysfunction and apathy-like behavior. These results indicate that TDP-43 can exert direct pathological effects in the hypothalamus, which may contribute to the development of the non-motor phenotype in TDP-43 proteinopathies.

**Supplementary Information:**

The online version contains supplementary material available at 10.1186/s40478-025-02018-8.

## Introduction

TAR DNA-binding protein of 43 kDa (TDP-43) proteinopathies including amyotrophic lateral sclerosis (ALS), frontotemporal dementia (FTD), and Huntington disease (HD) are neurodegenerative disorders characterized by TDP-43 pathology [16]. The TDP-43 protein encoded by the *TARDBP* gene is a ubiquitously expressed DNA and RNA binding protein mainly localized in the cell nucleus [13, 14, 65]. TDP-43 forms inclusions in the brain of almost all individuals with ALS and in around half of individuals with FTD [5, 57, 59, 62, 63, 76]. These inclusions are both nuclear and cytoplasmic, and TDP-43 has been found to be ubiquitinated as well as to undergo nuclear-to-cytoplasmic mislocalization leading to both gain-of function and loss of function mechanisms [5, 37, 63]. Interestingly, TDP-43 also forms inclusions in HD and interacts with mutant huntingtin, the disease-causing protein of HD [33, 64, 72, 73, 78].

In addition to TDP-43 pathology, these neurodegenerative diseases share early manifestation of non-motor features including psychiatric and metabolic changes that may be caused by pathology of the hypothalamus, a brain region critical for regulating these functions [2, 3, 8, 17, 34, 38, 66, 69, 74, 92]. Hypothalamic atrophy is observed in these disorders, affecting the entire hypothalamus in ALS/FTD and the lateral tuberal and paraventricular nuclei in HD [21, 28, 30, 34, 47, 52, 69, 74, 82, 86]. Selective loss of hypothalamic neurons expressing the emotion and metabolism regulating neuropeptides oxytocin and hypocretin (orexin) has been identified in both ALS and HD [6, 28–30, 68]. Furthermore, TDP-43 inclusions have been found in hypothalamic nuclei in post-mortem tissue from individuals with ALS and FTD, including in hypocretin and oxytocin-expressing neurons [21]. The number of melanin-concentrating hormone (MCH) neurons has been shown to be reduced in ALS, but not in clinical HD [6, 8, 12]. Loss of MCH neurons have recently been linked to the early manifestation of sleep disturbances in presymptomatic ALS gene carriers [35]. Hence, this selective vulnerability of hypothalamic neuronal populations may contribute to the early onset of non-motor features in these disorders. However, direct causal relationships between hypothalamic pathology in these disorders and disease manifestations remain to be fully established. Hypothalamic atrophy has been shown to correlate to reduced body mass index (BMI), abnormal eating habits as well as behavioral changes such as apathy in cases with ALS and FTD using MRI analyses [34, 82].

Hypothalamic pathology may be an important part of the early development of neurodegenerative disorders. We hypothesize that TDP-43 exerts direct pathological effects in the hypothalamus leading to the development of behavioral and metabolic changes. In the present study, we investigated whether there is direct causality between overexpression of TDP-43 in the hypothalamus and the development of neuropathology as well as metabolic and behavioral dysfunction. We generated an adeno-associated viral (AAV) vector expressing wild-type human TDP-43 under the synapsin promoter and delivered it bilaterally to the hypothalamus of FVB/N mice. We found that TDP-43 can directly elicit pathological effects in the hypothalamus, relevant to the early clinical phenotype of TDP-43 proteinopathies.

## Materials and methods

### Animals

Female FVB/N mice (Janvier Labs) were housed in cages of 2–5 animals and maintained in a 12 h light/dark cycle with *ad libitum* access to food and water. The experimental procedures were approved by the Malmö/Lund Regional Ethical Committee, Sweden (Permit: 17113/2022). In accordance with this ethical permit, mice needed to be sacrificed if a humane endpoint is reached. These humane endpoints were defined as manifestation of complete immobility, eye squinting and raggedness of fur. The requirements were consistently monitored across all experiments to ensure animal welfare. Only mice in Experiment II reached the humane endpoint prior to the scheduled experiment endpoint.

### Experiment I

We constructed an AAV vector designed to express wild-type human TDP-43 under the human synapsin 1 promotor (AAV-TDP43). To verify overexpression of TDP-43 and to determine the dose-dependent effect of the AAV-TDP43 vector on hypothalamic neuropathology, mice were bilaterally injected with three doses of the AAV vector (5.0 × 10^13^ (*n* = 6); 1.0 × 10^14^ (*n* = 6); 2.5 × 10^14^ (*n* = 6) genome copies (gc)/ml)) in the hypothalamus at 6 weeks of age. Two uninjected mice were used as controls. Mice were perfused 9 weeks post-injection.

### Experiment II

To further evaluate the effects on neuropathology following TDP-43 overexpression in the hypothalamus, 10 mice were bilaterally injected in the hypothalamus with the AAV-TDP43 vector (2.5 × 10^14^ gc/ml) at 6 weeks of age and 8 uninjected mice of the same age served as controls. Injected mice were required to be euthanized 6 or 10 weeks post-injection (*n* = 5/timepoint) according to humane endpoints defined in the ethical permit 17113/2022 including the manifestation of immobility, eye squinting and raggedness of fur. The uninjected controls were sacrificed at 18 weeks of age. Brains were neuropathologically examined. To confirm that the neuropathological effects following overexpression of TDP-43 was not due to simply overexpression of any protein, AAV vectors expressing green fluorescent protein (GFP) (AAV-GFP) or a fragment of mutant huntingtin (mHTT) with a 79 polyglutamine tract (AAV-mHTT) were used at the same titer (2.5 × 10^14^ gc/ml). These vectors have been produced and described previously [42]. Mice were bilaterally injected with either AAV-GFP (*n* = 5) or AAV-mHTT (*n* = 4) in the hypothalamus at 6 weeks of age and sacrificed at 10 weeks post-injection.

### Experiment III

To investigate effects on metabolism, behavior and molecular properties of TDP-43 following TDP-43 overexpression in the hypothalamus, 19 mice were bilaterally injected in the hypothalamus with the AAV-TDP43 vector (1.0 × 10^14^ gc/ml) and 20 mice with vehicle (10 ppm poloxamer-188 in phosphate-buffered saline (PBS)). Mice were weighed biweekly throughout the experiment. Mice underwent behavioural testing at 18 weeks post-injection using open field and elevated plus maze, at 19 weeks post-injection using the rotarod test and at 20 weeks post-injection using the forced swim test. Fat composition was determined with dual energy X-ray absorptiometry (DEXA) scan at 20 weeks post-injection. Mice were perfused at 21 weeks post-injection. Brains were immunohistochemically processed for TDP-43 and targeting of neuropeptide neuronal populations. Blood was collected from the right ventricle between 8:00 and 15:00 without prior fasting, and serum was saved for glucose measurements.

### Experiment IV

To determine effects on food intake, nesting behavior, neuroinflammation and gene expression following TDP-43 overexpression in the hypothalamus, 16 mice were bilaterally injected in the hypothalamus with the AAV-TDP43 vector (1.0 × 10^14^ gc/ml) and 16 control mice were injected with vehicle. Mice were weighed biweekly throughout the experiment. Prior to the 8-week post-injection assessments, one AAV-TDP43-injected mouse unexpectedly died, leading to the exclusion of its cage from nesting and food intake measurements. Mice were sacrificed at 10 weeks post-injection and hypothalami were dissected for qRT-PCR and RNA sequencing analyses.

### AAV vector production and stereotactic delivery

Recombinant AAV vector plasmids were derived from a previously described double-transfection method [84]. The AAV-TDP43 vector was designed to express the coding sequence of wild-type human *TARDP* gene. The design of the vector including the choice of AAV serotype as well as the delivery method was based on previous experiments expressing transgenes into the hypothalamus models for HD [42]. We also used two previously described AAV-vectors, the AAV-mHTT vector that expresses the first 853 amino acids of the N-terminal fragment of huntingtin with 79 polyglutamines and the AAV-GFP vector expressing GFP [42]. In all vectors, the transgene sequences were regulated by the human synapsin 1 promoter and flanked with simian virus 40 polyadenylation signal sequence between the inverted terminal repeat from AAV2 genome and packaged in a capsid from AAV5 genome. All vectors were dissolved with 10 ppm poloxamer-188 in PBS and titers were estimated by TaqMan quantitative polymerase-chain reaction. The AAV-TDP43 titers were 5.0 × 10^13^, 1.0 × 10^14^ and 2.5 × 10^14^ gc/ml. The AAV-mHTT and AAV-GFP titers were 2.5 × 10^14^ gc/ml. Stereotactic delivery of 0.5 µl/side of the AAV vectors or vehicle was performed as previously described [10] at the following coordinates: anterior/posterior= -0.7 mm, medial/lateral: ± 0.55 mm, ventral/dorsal = -5.2 mm. The stereotactic coordinates were calculated relative to the bregma and dura.

### Immunohistochemistry

#### Peroxidase-based immunohistochemistry

Mice were sacrificed using sodium pentobarbital (~ 150 mg/kg i.p.) and transcardially perfused with first saline and then pre-cooled 4% paraformaldehyde (PFA) solution. Brains were removed, and then post-fixed for 24 h in 4% PFA and placed in 25% sucrose for cryoprotection. Brains were sectioned frozen in the coronal plane at a thickness of 30 µm in six series. The sections were processed as previously described [10]. Primary antibodies were raised against TDP-43 (1:100 000, made in rabbit, 10782-2-AP, ProteinTech), oxytocin (1:4000, made in rabbit, H-051-01, Phoenix Pharmaceuticals), hypocretin (1:4000, made in rabbit, H-003-30, Phoenix Pharmaceuticals), vasopressin (1:10 000, made in rabbit, AB1565, Merck Milipore), MCH (1:20 000, made in rabbit, H-070-47, Phoenix Pharmaceuticals), huntingtin (1:500, made in goat, SC-8767, Santa Cruz Biotechnology), GFP (1:20 000, made in chicken, ab13970, Abcam), ubiquitin (1:2000, made in rabbit, Z0458, DakoCytomation), GFAP (1:10 000, made in rabbit, Z0334, DakoCytomation) and Iba1 (1:1000, made in rabbit, 019-19741, FUJIFILM Wako Pure Chemical Corporation). Sections were labelled with suitable secondary antibody (1:200, anti-rabbit, anti-goat or anti-chicken) and visualised with 3,3’-diaminobenzidine (DAB) as chromogen using hydrogen peroxidase. Lastly, sections were mounted on chromatin-gelatin coated glass slides and dehydrated in an ethanol and xylene series and coverslipped with DPX (Sigma Aldrich).

For cresyl violet (CV) staining, slide-mounted sections were first dehydrated in an ethanol and xylene series. Sections were then briefly dipped in deionized water followed by lithium carbonate solution (0.5 g lithium carbonate in 1000 ml deionized water) for 30 s, 70% ethanol for 30 s, deionized water for 30 s and 0.5% CV solution for 1 min with 20 drops of 10% acetic acid were added per 100 ml CV solution. Slides were again dehydrated through an ethanol series.

#### Fluorescence immunohistochemistry

To confirm targeting of AAV-mediated TDP-43 overexpression in hypocretin, MCH, oxytocin or vasopressin neurons, brain sections were blocked in 0.25% triton X-100 in potassium phosphate-buffered saline (KPBS-T) containing 5% donkey serum for 1 h. Subsequently, the sections were incubated overnight using antibodies against oxytocin (1:2000, made in rabbit, H-051-01, Phoenix Pharmaceuticals), hypocretin (1:2000, made in rabbit, H-003-30, Phoenix Pharmaceuticals), vasopressin (1:1000, made in rabbit, AB1565, Merck Milipore) and MCH (1:2000, made in rabbit, H-070-47, Phoenix Pharmaceuticals) in KPBS-T containing 5% donkey serum. Sections were labelled with Alexa Fluor 647-conjugated donkey anti-rabbit secondary antibody (1:200, Jackson Laboratories), followed by an additional blocking step of 5% rabbit serum in KPBS-T for 2 h. Samples were then incubated overnight with biotin-conjugated TDP-43 (1:200, made in rabbit, Biotin-10782, ProteinTech), followed by Alexa Fluor 488-conjugated streptavidin (1:200, Jackson Laboratories). For analyses of intracellular TDP-43 localisation, the samples were incubated with a primary antibody against TDP-43 (1:1000, made in rabbit, 10782-2-AP, Proteintech) followed by Alexa Fluor 647-conjugated secondary antibody (1:200, Jackson Laboratories). After secondary antibody incubation, sections were stained with Hoechst 33342 (1:1000, 62249, Thermo Fisher) for 5 min. All sections were mounted on chromatin-gelatin coated glass slides and coverslipped using Vectashield mounting medium (Vector Laboratories).

#### Image acquisition

Brightfield and fluorescence image acquisition was performed using a Zeiss Axio Imager M2 microscope (Zeiss, Göttingen, Germany) equipped with apochromatic objectives (5x/0.15 NA, 10x/0.3 NA, 20x/0.8 NA, and 40x/1.3 NA oil). Brightfield images were acquired with a Zeiss Axiocam 305 color camera, and fluorescent images with a Zeiss Axiocam 805 monochrome camera. For each figure, images were acquired with consistent settings, and contrast was optimized by adjusting the histogram using the Zeiss Zen 3.8 software. Image panels were created and edited using Affinity Designer 1.10.

### Cell quantification and hypothalamic volume measurements

Brain sections immunohistochemically processed with antibodies against hypocretin, MCH, oxytocin, and vasopressin were analyzed on a Nikon Eclipse 80i microscope using a 60x plan-apochromat 1.4 N.A. oil objective. The VIS software (Visiopharm) was used for all analyses. The experimenter was blinded to the experimental groups for all cell quantification and volume measurements. All hypocretin- and MCH-expressing neurons were counted bilaterally in the hypothalamus. Oxytocin- and vasopressin-expressing neurons in or bordering the paraventricular nucleus were counted bilaterally. Hypocretin- and MCH-expressing neurons were located around bregma −1.06 to −2.30 mm, and oxytocin- and vasopressin-expressing neurons at bregma −0.46 to −1.22 mm. The total number of neurons was estimated by total cell count multiplied with the number of series (6).

Hypothalamic volume was measured from sections spanning bregma −0.35 mm to −2.18 mm. The hypothalamus was outlined along the third ventricle to the optic tract. In experiment II, images were captured on a Nikon Eclipse 80i (4x/0.1 N.A). The hypothalamus was measured bilaterally using VIS software. In experiment III, images were captured with the Zeiss PrimoStar microscope (4x/0.1 N.A). The hypothalamus was measured unilaterally using the ImageJ 1.53t software. Volume was calculated by multiplying the outlined hypothalamic area by section thickness (30 μm), the number of series (6), and, in experiment III, the number of hemispheres (2).

### RNA sequencing

RNA isolation was performed on frozen hypothalami from mice 10 weeks post-injection in experiment IV using the RNeasy Mini Kit (Qiagen), according to the manufacturer’s recommendations, except for the omission of β-mercaptoethanol in the buffers (*n* = 6/group). Disruption of samples was performed using the TissueLyser II (Qiagen) for 2 × 2 min at 30 Hz using 3 mm Tungsten Carbide Beads and RLT buffer, and the inclusion of the RNAse-Free DNase Set. Upon completion of the protocol, the RNA was eluted in 35 µl of RNase-free water. The concentration of RNA was measured using the Qubit Fluorometric Quantitation and RNA Broad-Range Assay (Thermo Fisher Scientific). RNA integrity number (RIN) was determined using the Fragment Analyzer 5300 and Fragment Analyzer RNA kit (Agilent Technologies) and showed good integrity (RIN > 8.8). For library preparation, the mRNA fraction was enriched using polyA capture from 100 ng of total RNA using the NEBNext Poly(A) mRNA Magnetic Isolation Module (NEB). Subsequently, libraries were prepared using the NEB Next Ultra II Directional RNA Library Prep Kit for Illumina and NEBNext UDI UMI (NEB) following the manufacturer’s instructions. To minimize technical batch effects, library preparations were performed using the Liquid Handler Biomek i7 (Beckman Coulter). The library molarity was determined by measuring the library size (approximately 400 bp) using the Fragment Analyzer 5300 and the Fragment Analyzer DNA HS NGS fragment kit (Agilent Technologies), and the library concentration (> 2 ng/µl) using Qubit Fluorometric Quantitation and dsDNA High Sensitivity Assay (Thermo Fisher Scientific). The libraries were denatured according to the manufacturer’s instructions, diluted to 150 pM, and sequenced as paired-end 100 bp reads on an Illumina NovaSeq 6000 (Illumina). Sequencing was aimed at achieving a depth of > 25 million clusters per sample. The read quality of RNA-seq data in fastq files was assessed using ngs-bits (2023_11) to identify sequencing cycles with low average quality, adaptor contamination, or repetitive sequences from the PCR amplification. Reads were aligned using STAR 2.7.11b [23] to the GRCm39 and alignment quality was analyzed using ngs-bits and visually inspected in the Integrative Genome Viewer 2.15.4. Normalized read counts for all genes were obtained using Subread 2.0.6 and edgeR 3.42.4. Raw expression values were available for 55 416 genes in 12 samples. Raw gene expression was filtered by a minimum expression value of 1 count per million (cpm) in at least two samples. The filtered data contained the expression values for 16 189 genes. The distribution of the logarithmized cpm-normalized expression values showed similar characteristics for all samples. Based on the filtered dataset, samples were investigated with respect to their pairwise similarity. Differential gene expression analysis was conducted based on the filtered gene expression dataset. For each gene, gene expression log2 fold changes were computed, and was statistically tested for significance using edgeR generalized linear model and the quasi-likelihood method.

### Quantification of exon skipping

Exon skipping was quantified using Integrative Genome Viewer 2.15.4, with sashimi plots of exon junctions displayed for each sample. The number of reads that bypassed the exon of interest was divided by the mean of the reads for exon junctions preceding and following the exon of interest. Junctions with fewer than five reads were excluded from data presentation.

### qRT-PCR

To confirm hypothalamic TDP-43 overexpression, mRNA levels of *TARDBP/Tardbp* were analyzed by quantitative real-time PCR. Reverse transcription was conducted using a QuantiTect Reverse Transcription Kit (Qiagen) with 250 ng of RNA input and gDNA digestion. Gene-specific primers were designed to amplify both human *TARDBP* (NM_007375.4) and mouse *Tardbp* (NM_145556.4) using Primer-BLAST [91]. with an amplicon size of 75–150 bp and melting at 60 °C. An exon junction was incorporated to mitigate potential signals related to genomic DNA, resulting in the forward primer AAGGAATTCTGCATGCCCCA and reverse primer TGTTTTCTGGACTGCTCTTTTCAC. For reference genes, primers targeting the mouse *Gapdh* forward primer TGTCCGTCGTGGATCTGAC and reverse primer CCTGCTTCACCACCTTCTTG, as well as the mouse *Pdhb* forward primer GTAGAGGACACGGGCAAGAT and reverse primer TGAAAACGCCTCTTCAGCA were employed. Quantitative PCR was performed using the QuantiTect SYBR Green RT-PCR Kit (Qiagen) with a master mix of 10 µl and including 1 µL of the resulting cDNA samples diluted to 1:20. All real-time PCR were conducted using a LightCycler 480 Instrument II (Roche). The thermal cycling conditions were as follows: initial denaturation at 95 °C for 15 min, followed by 45 cycles of 94 °C for 15s, 55 °C for 30s and 72 °C for 30s. A melting curve was obtained from 65 °C to 95 °C with a Ramp Rate of 0.04 °C/s and 15 acquisitions per °C. A standard curve of all mixed cDNA was generated using a raw dilution, and the cDNA was diluted to 1:5, 1:20, 1:80, and 1:320 to correct for qPCR efficiency. Experiments were performed in duplicate for each data point, and the relative quantification of gene expression was determined using the efficiency-corrected 2-ΔΔCt method [58].

### Body fat and serum glucose concentration

To measure body fat percentage at 20 weeks post-injection, mice were anaesthetized with a 2% isoflurane/N_2_O mixture and individually scanned using the DEXA Lunar PIXImus2 scanner (Lunar Corporation). Body fat percentage was calculated using PIXImus2 2.10 software. Serum glucose concentration was determined by the glucose oxidase method coupled to a peroxidase reaction to colorimetrically detect H_2_O_2_ formation [24].

### Behavioural procedures

#### Open field and elevated plus maze

The open field test was used to assess motor activity and elevated plus maze to assess anxiety-like behaviour of mice bilaterally injected with AAV-TDP43 or vehicle at 18 weeks post-injection as previously described [10, 41, 67]. Mice were recorded for 1 h in the open field test and 5 min in elevated plus maze using the Ethovision XT 13 software. Mice were randomly assigned to a trial using a random number generator. Mice were moved into testing area 1 h before testing and the experiments were performed between 8:00–13:00.

#### Rotarod

An accelerating rotarod device (Ugo Basile S.R.L.) was used to evaluate motor activity and balance in mice at 19 weeks post-injection. Prior to the rotarod test, mice underwent three 2-minute-long training sessions at a constant speed of 5 revolutions per minute (rpm) over 3 consecutive days, with 2-hour inter-session intervals. If the mice fell, they were returned to the rod. The rotarod test consisted of accelerating rotation from 5 rpm to 40 rpm over 5 min. The test ended if the mouse fell off the rod or if 5 min had passed. The test was repeated three times with 2-hour intersession intervals. The order in which the mice were tested was randomized and the mice were transferred to the testing area 1 h before testing. The tests were performed between 8:00–13:00.

#### Forced swim test

The forced swim test was used to assess depressive-like behaviour as immobility in mice at 20-weeks post-injection as described previously [20, 41]. Mice were recorded for 5 min and the time spent immobile during the last 3 min was manually scored by an experimenter blinded to the treatment groups. The order in which the mice were tested was randomized and the mice were transferred to the testing area 1 h before testing. The tests were performed between 8:00–13:00.

### Nesting and food intake

Nesting and food intake were assessed over a 4-day period (with measurements at day 0 and 4) at 4- and 8-weeks post-injection of AAV-TDP43 or vehicle at the same time of the day. Mice of the same treatment group were pair-housed with a 2-day acclimatizing period with *ad libitum* access to food and water. Food intake per cage over four days was determined by calculating the difference in total food weight between day 0 and day 4. At the start of the nesting behavior analysis, 7–8 g of intact nesting material was provided in each cage. The experimenter was blinded to the experimental groups during nesting behavior assessment at both time points. Nesting ability was assessed based on a modified scoring system from Gaskill et al. [32]. Briefly, a score of 0 was assigned if the material appeared untouched, a score of 1 was given for manipulated material without starting to cup or form a dome, a score of 2 indicated manipulated material and a partially formed nest with a developing cup, and a score of 3 indicated complete manipulation of material with a fully cupped dome.

### Statistics

Data was analysed using the independent samples Mann-Whitney U test in SPSS 18.0, and p-values were corrected for multiple comparisons using Bonferroni when applicable. Spearman’s rank test was used for correlation analyses. Statistical significance was set at *p* < 0.05 (exact, 2-tailed). Graphs were generated using GraphPad Prism 10.2. Data are presented as median with range.

## Results

### AAV-vector mediated expression of human wild-type TDP-43 in the hypothalamus leads to neuropathology in a dose-dependent fashion

**Fig. 1:**
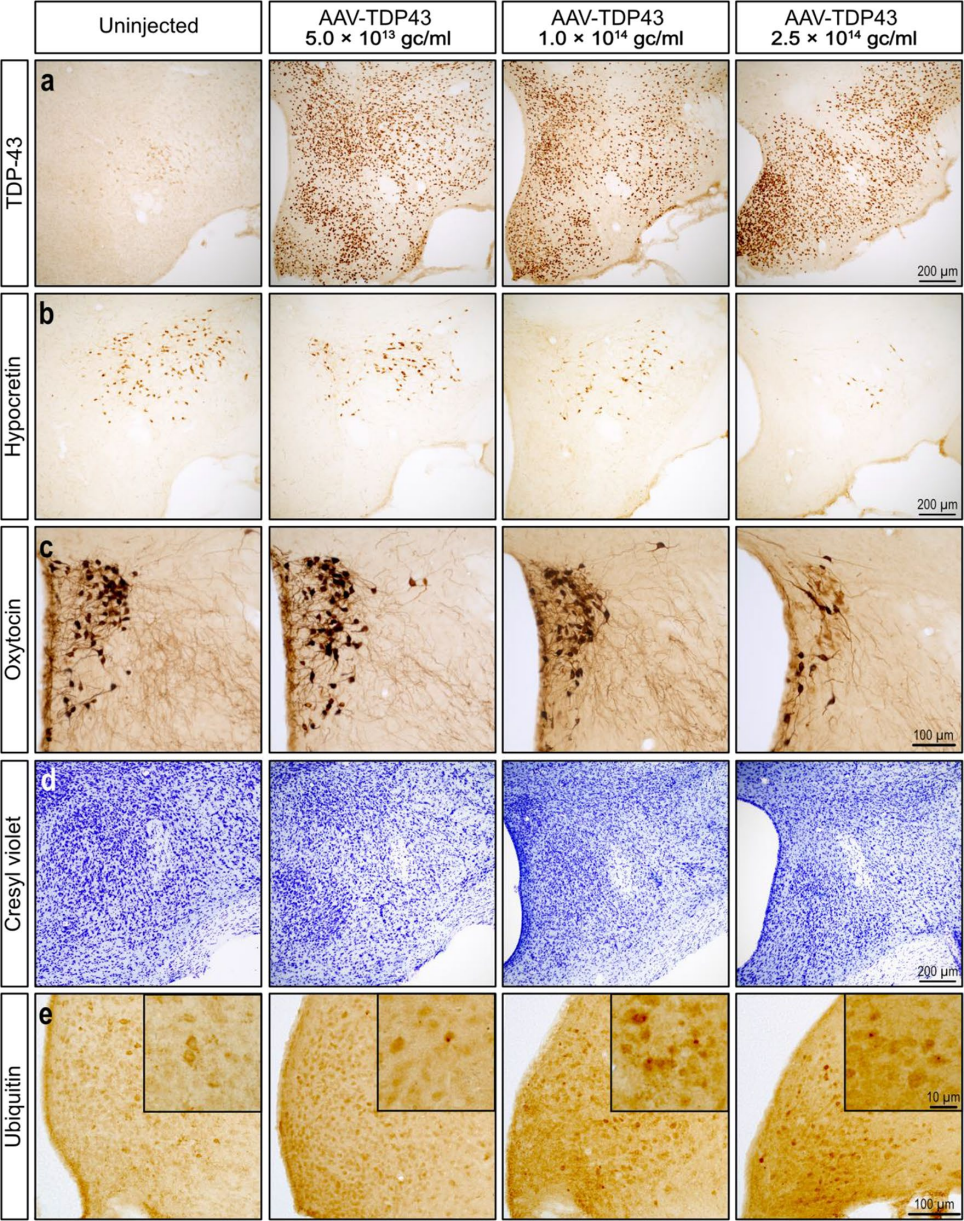
Overexpression of TDP-43 induces neuropathology in the hypothalamus in a dose-dependent fashion. Mice were injected with 5.0 × 10^13^ genome copies (gc)/ml, 1.0 × 10^14^ gc/ml, or 2.5 × 10^14^ gc/ml titers of the AAV-TDP43 vector. **a**. Immunoreactivity for TDP-43 was comparable in the hypothalamus across all injected groups. The number of neurons immunoreactive for hypocretin (**b**) and oxytocin (**c**) appeared to decrease in a dose-dependent fashion. **d**. Neuronal loss was observed in the lateral hypothalamic area only in mice injected with the highest dose. **e**. Mice injected with the AAV-TDP43 vectors showed increased ubiquitin immunoreactivity in a dose-dependent fashion with ubiquitin-immunopositive inclusions

We generated an AAV vector to express wild-type human TDP-43 using the same AAV-vector design and the same neuronal specific synapsin promotor used in Hult et al. [42]. To confirm overexpression of TDP-43 and to begin investigating the potential neuropathological effects, we first performed stereotaxic bilateral injections of AAV-TDP43 at three different titers: 5.0 × 10^13^ gc/ml (*n* = 6), 1.0 × 10^14^ gc/ml, (*n* = 6), or 2.5 × 10^14^ gc/ml (*n* = 6) into the hypothalamus of adult female FVB/N mice. TDP-43 overexpression in the hypothalamus led to widespread TDP-43 immunoreactivity throughout the hypothalamus across all groups, including in the paraventricular nucleus and the lateral hypothalamic area, which contain hypocretin- and oxytocin-expressing neurons that are significantly affected in postmortem ALS cases (Fig. 1a) [28]. TDP-43 immunoreactivity was concentrated to the hypothalamus after the AAV-delivery, but single cells overexpressing TDP-43 could be observed in other brain regions, including the cerebral cortex, striatum, and hippocampus (Supplementary Fig. 1). Qualitative analysis showed a decrease in the number of hypocretin- and oxytocin-expressing neurons in a dose-dependent fashion, with the most pronounced loss in highest titer group (Fig. 1b-c). Neuronal loss was confirmed by analysis of CV-stained sections (Fig. 1d). Additionally, ubiquitin-immunoreactivity was increased in a dose-dependent fashion with ubiquitin-immunoreactive inclusions present in mice overexpressing TDP-43 (Fig. 1e). Consistent with previous findings using the same AAV-vector design [42], we confirmed that AAV-mediated TDP-43 overexpression targets hypothalamic neurons expressing hypocretin, MCH, oxytocin, and vasopressin (Supplementary Fig. 2).

As TDP-43 overexpression in neurons could lead to non-cell autonomous effects on glial cells such as astrocytes and microglia triggering neuroinflammation [39] we investigated effects on glial neurofibrillary protein (GFAP) and ionized calcium binding adaptor molecule 1 (Iba1) immunoreactivity in mice overexpressing TDP-43 (*n* = 3) and vehicle-injected mice (*n* = 3). We observed increased GFAP immunoreactivity in mice overexpressing TDP-43 compared to vehicle-injected control mice. Furthermore, Iba1-immunoreactive cells in mice overexpressing TDP-43 showed distinct morphological changes, including increased and thickening of branches. These findings suggest astrogliosis and microglia activation indicative of an inflammatory response to TDP-43 overexpression (Supplementary Fig. 3).

### Overexpression of TDP-43 causes hypothalamic atrophy with selective loss of hypocretin-, oxytocin- and melanin-concentrating hormone-expressing neurons

**Fig. 2:**
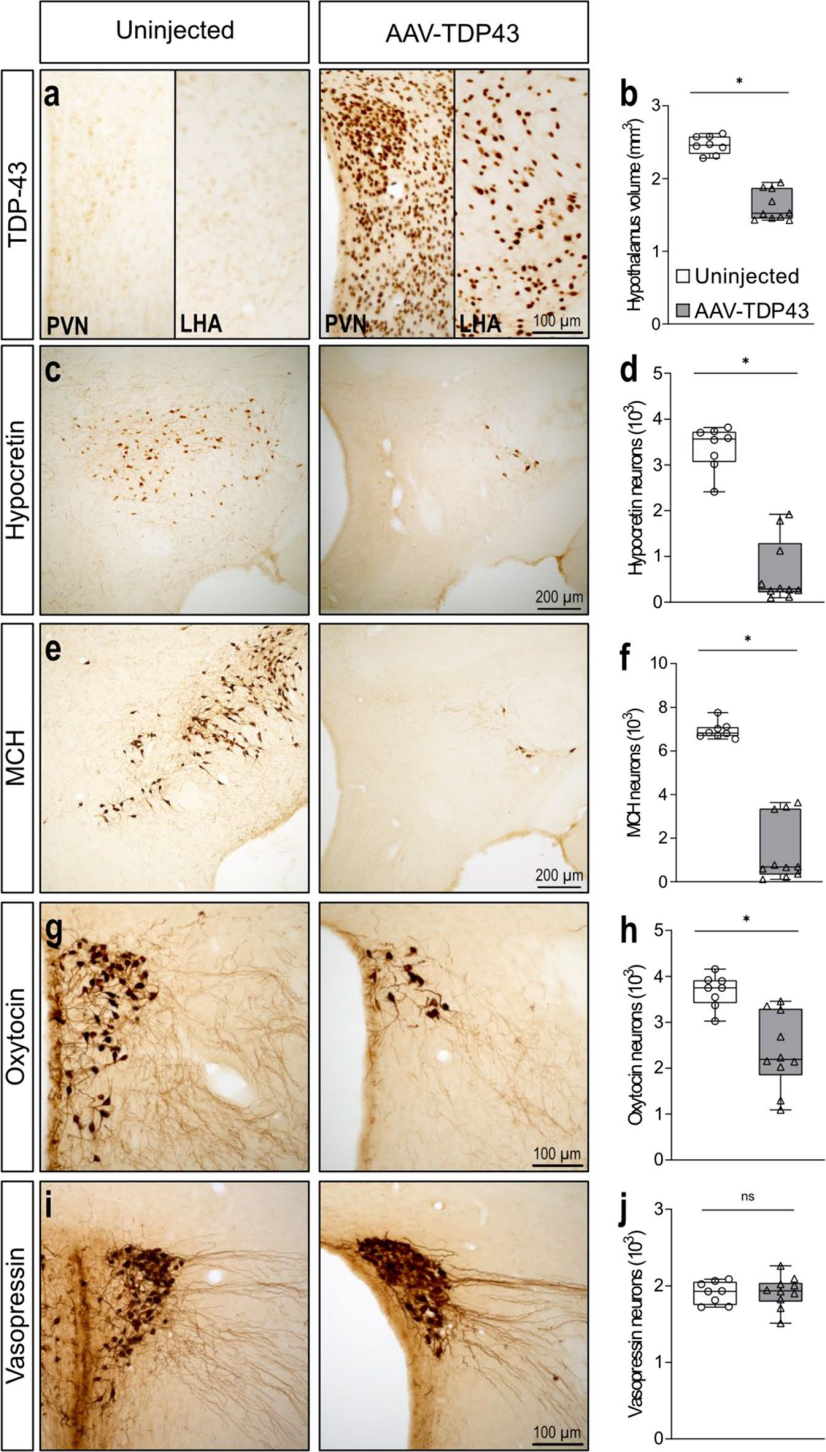
TDP-43 overexpression causes hypothalamic atrophy with selective loss of hypocretin-, oxytocin- and melanin concentrating hormone (MCH)-expressing neurons. **a**. TDP-43 immunoreactivity was detected in the hypothalamus in mice injected with AAV-TDP43, including in the paraventricular nucleus (PVN) and lateral hypothalamic area (LHA), which contain neurons expressing oxytocin and vasopressin as well hypocretin and MCH, respectively. **b**. Mice overexpressing TDP-43 in the hypothalamus displayed reduced hypothalamic volume compared to uninjected control mice (*p* < 0.001). **c**-**d**. Loss of hypocretin-expressing neurons was present in mice overexpressing TDP-43 in the hypothalamus compared to control mice (*p* < 0.001). **e**-**f**. A similar pattern was observed in MCH-expressing neurons with significant reductions in mice overexpressing TDP-43 (*p* < 0.001). **g**-**h**. The number of oxytocin-expressing neurons was reduced in mice overexpressing TDP-43 compared to control mice (*p* = 0.001). **i**-**j**. We observed no loss of vasopressin-expressing neurons. The data are presented as box-and-whisker plots with whiskers representing minimum and maximum values, * = *p* < 0.01 (2-tailed). Statistical analyses were performed using a Mann-Whitney U test

To further investigate the effects on neuropathology following TDP-43 overexpression in the hypothalamus, quantitative analyses of the hypothalamic volume and number of neuropeptide expressing neurons were performed in another cohort of mice bilaterally injected in the hypothalamus with the AAV-TDP43 vector (2.5 × 10^14^ gc/ml) and 8 uninjected mice of the same age that served as controls (Fig. 2a). The hypothalamic volume was reduced by 38% (1.5 mm^3^, 1.4-1.9 mm^3^, *p* < 0.001, *n* = 10) in mice overexpressing TDP-43 compared to uninjected control mice (2.5 mm^3^, 2.3-2.6 mm^3^, *n* = 8) (Fig. 2b). Mice overexpressing TDP-43 exhibited a 92% loss of hypocretin-expressing neurons (291, 96-1920, *p* < 0.001, *n* = 10) compared to control mice (3567, 2412-3816, *n* = 8, Fig. 2c-d). Similarly, mice overexpressing TDP-43 exhibited a 90% loss of MCH-expressing neurons (681, 114-3636, *p* < 0.001, *n* = 10) compared to control mice (6819, 6552-7752, *n* = 8) (Fig. 2e-f). Mice overexpressing TDP-43 also exhibited a 42% loss of oxytocin-expressing neurons (2193, 1092-3456, *p* = 0.001, *n* = 10) compared to control mice (3750, 3024-4158, *n* = 8) (Fig. 2g-h). In contrast, vasopressin-expressing neurons were not affected in mice overexpressing TDP-43 (*p* = 1.000, *n* = 10) compared to control mice (*n* = 8, Fig. 2i-j). Interestingly, hypothalamic atrophy, selective loss of neurons expressing hypocretin, MCH and oxytocin neurons and sparing of vasopressin-expressing neurons as observed in mice overexpressing TDP-43 are consistent with previous studies in postmortem human ALS cases [12, 28].

In order to confirm that the effects on the hypothalamus were not due to overexpression of any protein, a similar experiment was performed where mice received hypothalamic injections of AAV vectors expressing either GFP (AAV-GFP) or a large fragment of mutant huntingtin (AAV-mHTT) at the same titer used in experiment II (2.5 × 10^14^ gc/ml). At 10 weeks post-injection, mice overexpressing GFP showed no overt neuropathology of hypocretin- MCH-, or oxytocin-expressing neurons (Supplementary Fig. 4). As expected, mutant huntingtin overexpression resulted in decreased numbers of hypocretin-expressing neurons, consistent with prior results [42]. These results confirm that the neuropathology in the hypothalamus after overexpression of TDP-43 is not simply a result of overexpression of any protein.

### TDP-43 overexpression in the hypothalamus induces obesity and hyperglycaemia in mice

**Fig. 3:**
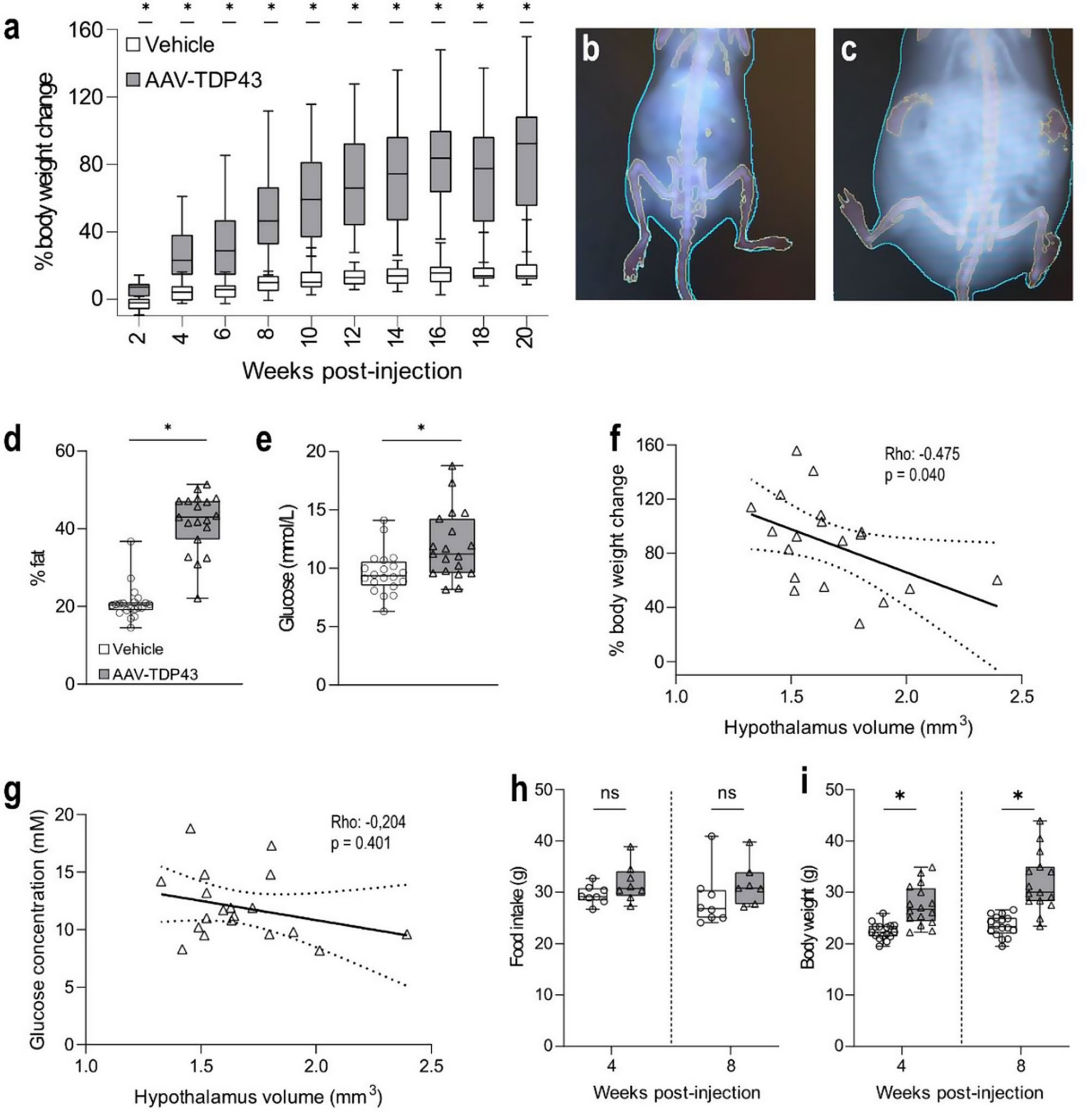
TDP-43 overexpression in the hypothalamus induces obesity and hyperglycaemia in mice. **a**. Mice overexpressing TDP-43 in the hypothalamus gained more weight compared to control mice injected with vehicle starting from 2 weeks post-injection. Dual energy X-ray absorptiometry scans of vehicle-injected control (**b**) and AAV-TDP43-injected mice (**c**) revealed that mice overexpressing TDP-43 had higher body fat percentage than control mice (*p* < 0.001) (**d**). e. We observed a significant increase in serum glucose levels in mice overexpressing TDP-43 compared to controls (*p* = 0.003). Spearman’s rank correlation test revealed a negative correlation for hypothalamic volume and percent body weight change (**f**), but no significant correlation for hypothalamic volume and serum glucose levels (**g**). In a separate cohort, mice were pair-housed and food intake was measured after 4 days at 4 and 8 weeks post-injection. Hypothalamic TDP-43 overexpression did not affect food intake at 4 and 8 weeks post-injection (**h**), despite higher body weight (**i**, *p* < 0.001 at both time-points). Data are presented as box-and-whisker plots with whiskers representing minimum and maximum values, * = *p* < 0.01 (2-tailed). Statistical analysis was performed using Mann-Whitney U test (**a**, **d**-**e**, **h**-**i**) and adjusted for multiple-comparison using Bonferroni (**a**)

Hypothalamic overexpression of TDP-43 induced selective loss of neuropeptide-expressing neurons involved in the regulation of metabolism and emotion, including hypocretin, MCH and oxytocin. Therefore, we next wanted to investigate the effects of TDP-43 overexpression in the hypothalamus on metabolism and behaviour in mice. Mice injected with AAV-TDP43 gained more weight compared to control mice injected with vehicle starting from 2 weeks post-injection (*p* = 0.001 at 2 weeks post-injection and *p* < 0.001 at subsequent time-points, AAV-TDP43 *n* = 19, vehicle *n* = 20, Fig. 3a). At 20 weeks post-injection, mice injected with AAV-TDP43 had increased in body weight by 92% (28-156%) compared to 14% (9–47%) in mice injected with vehicle. At that time-point, mice overexpressing TDP-43 in the hypothalamus had more than doubled in body fat percentage (43%, 22-51%, *p* < 0.001, *n* = 19) compared to control (20%, 15-37%, *n* = 20, Fig. 3b-d).

We also detected a 20% increase in serum glucose levels in mice overexpressing TDP-43 (12.7 mmol/L, 9.4-20.1 mmol/L, *p* = 0.003, *n* = 19) compared to vehicle injected mice (10.7 mmol/L, 7.2-15.7 mmol/L, *n* = 20, Fig. 3e). Spearman’s rank correlation test revealed a negative correlation between hypothalamic volume and body weight gain in mice overexpressing TDP-43 (rho: -0.475, *p* = 0.040, *n* = 19, Fig. 3f). This indicates that mice with more pronounced hypothalamic atrophy exhibited greater body weight gain. There was no significant correlation for hypothalamus volume and serum glucose levels (rho: -0.204, *p* = 0.401, *n* = 19, Fig. 3g).

A separate cohort of pair-housed mice was used to assess food intake per cage at 4 and 8 weeks following injection. We found no change in food intake in mice overexpressing TDP-43 at 4 weeks post-injection compared to control mice (*p* = 0.161, *n* = 8 cages/group at 4 weeks), while a trend of increased food intake was observed in mice overexpressing TDP-43 at 8 weeks post-injection compared to control, although not statistically significant (*p* = 0.073, AAV-TDP43 *n* = 7 cages, vehicle 8 cages, Fig. 3h). Despite unchanged food intake, mice overexpressing TDP-43 had a higher body weight compared to control mice at both 4 and 8 weeks post-injection also in this cohort. Here, TDP-43 overexpressing mice had a median body weight of 26.7 g at 4 weeks (22.2-34.9 g, *p* < 0.001, *n* = 16) and 30.0 g at 8 weeks (23.4-43.9, *p* < 0.001, *n* = 15) post-injection compared to control mice of 22.3 g (19.5-25.9 g, *n* = 16) and 23.4 g, respectively (19.5-26.6 g, *n* = 16, Fig. 3i). Taken together, these results demonstrate that hypothalamic TDP-43 overexpression disrupts metabolic homeostasis, leading to obesity and hyperglycaemia. Increased body weight gain in mice overexpressing TDP-43 correlates with hypothalamic atrophy and appears to occur independent on food intake.

### Mice overexpressing TDP-43 in the hypothalamus display significantly reduced motor activity but maintain ability to swim

**Fig. 4:**
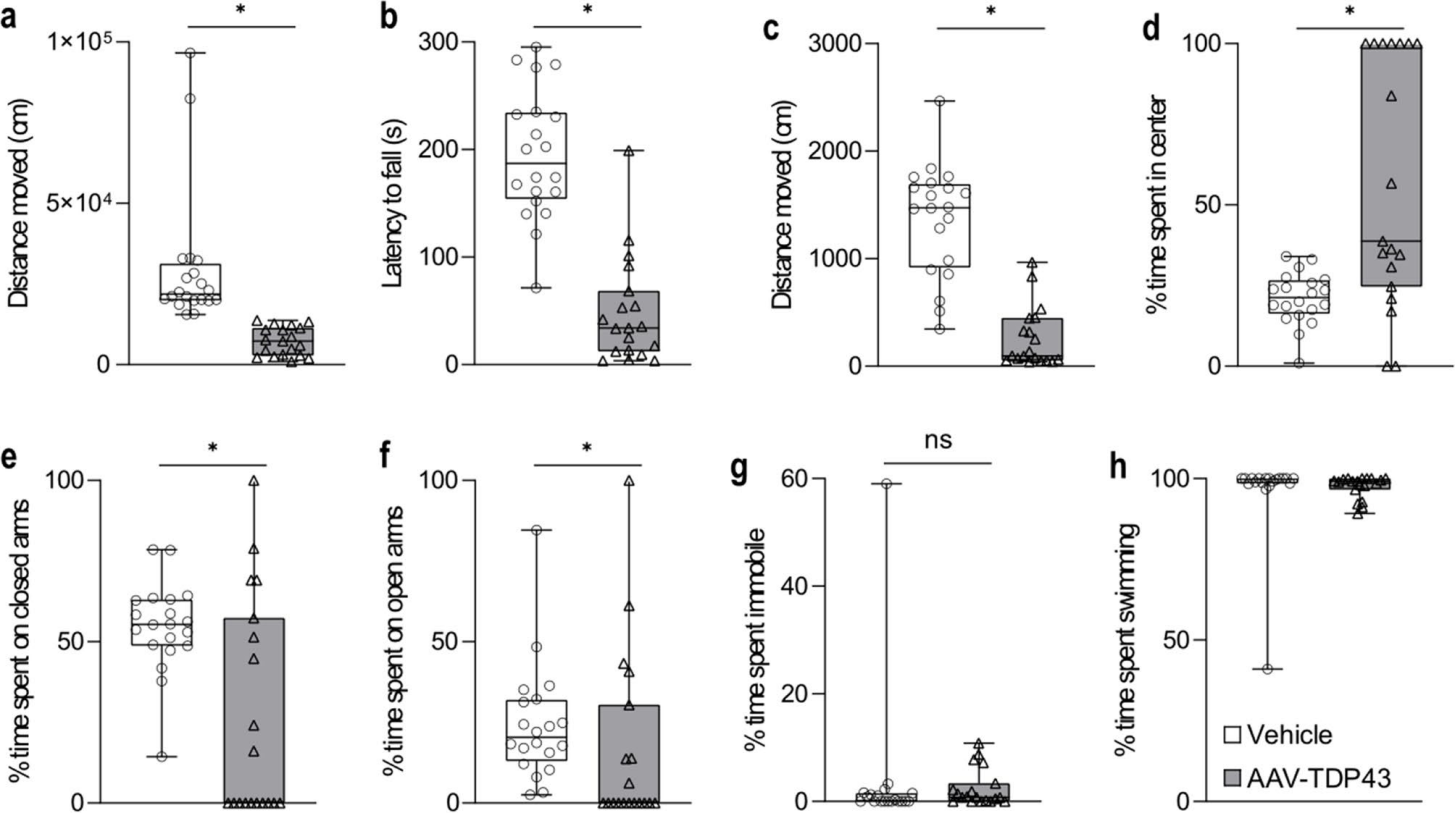
Mice overexpressing TDP-43 in the hypothalamus show significantly reduced motor activity but maintain ability to swim. **a** Mice overexpressing TDP-43 travelled a shorter distance in the open field test than control mice injected with vehicle (*p* < 0.001). **b**. The rotarod test revealed that mice overexpressing TDP-43 had a shorter latency to fall from the device than control mice (*p* < 0.001). Results from the elevated plus maze revealed that mice overexpressing TDP-43 in the hypothalamus also travelled shorter distances (**c**, *p* < 0.001), and that they spent more time in the center of the arena (**d**, *p* = 0.001) where the mice were placed at the start of the trial and less time on both closed (**e**, *p* = 0.011) and open arms (**f**, *p* = 0.012) compared to control mice. However, overexpressing TDP-43 does not limit swimming ability (**g**-**h**). Data are presented as box-and-whisker plots with whiskers representing minimum and maximum values, * = *p* < 0.05 (2-tailed). Statistical analyses were performed using Mann-Whitney U test

Motor impairment and psychiatric features, including anxiety, depression, and apathy, are seen in individuals with ALS/FTD and HD [25, 60]. To determine the effects of TDP-43 overexpression in the hypothalamus on motor activity and behavior, we first investigated motor activity using the open field test and rotarod test. In the open field test, mice overexpressing TDP-43 in the hypothalamus travelled a shorter distance by 67% (73.1 m, 9.9-137.4 m, *p* < 0.001, *n* = 19) compared to control mice injected with vehicle (218.5 m, 155.1-966.0 m, *n* = 20, Fig. 4a). Similarly, on the rotarod test, mice overexpressing TDP-43 exhibited a pronounced 82% decrease in latency to fall (34 s, 4-199 s, *p* < 0.001, *n* = 19) compared to controls (187 s, 71-295 s, *n* = 20, Fig. 4b). These results demonstrate that TDP-43 overexpression in the hypothalamus leads to decreased motor activity and may impair motor coordination in mice.

Next, we used the elevated plus maze and the forced swim test to assess the emotional phenotype in mice overexpressing TDP-43. In the elevated plus maze, mice overexpressing TDP-43 in the hypothalamus displayed reduced distance travelled by 93% (97 cm, 42-965 cm, *p* < 0.001, *n* = 19) compared to control mice (1473 cm, 345-2467 cm, *n* = 20, Fig. 4c). Mice overexpressing TDP-43 spent more time in the center of the arena (39%, 0-100%, *p* = 0.001), where mice were placed before start of the trial, compared to control mice (21%, 1-34%, Fig. 4d). Median time spent for mice overexpressing TDP-43 was 0% on closed arms (0-100%, *p* = 0.011) compared to control mice at 55% (14-79%, Fig. 4e) and 0% on open arms (0-100%, *p* = 0.012) compared to control mice at 20% (3-85%, Fig. 4f), suggesting that mice overexpressing TDP-43 display reduced exploratory behavior.

In the forced swim test, commonly used to assess depressive-like behavior, there was no difference in immobility between mice overexpressing TDP-43 and vehicle-injected mice, indicating no depressive-like behavior induced by TDP-43 overexpression in the hypothalamus (*p* = 0.181) (Fig. 4g). In fact, mice with TDP-43 overexpression spent most of the time swimming similar to control mice and thus TDP-43 overexpression does not seem to limit the ability to swim (Fig. 4h). Taken together, TDP-43 overexpression in mice leads to reduced motor activity and reduced exploratory behavior but this is not caused by inability to move as indicated by maintained swimming ability.

### Mice overexpressing TDP-43 in the hypothalamus develop apathy-like behavior

**Fig. 5:**
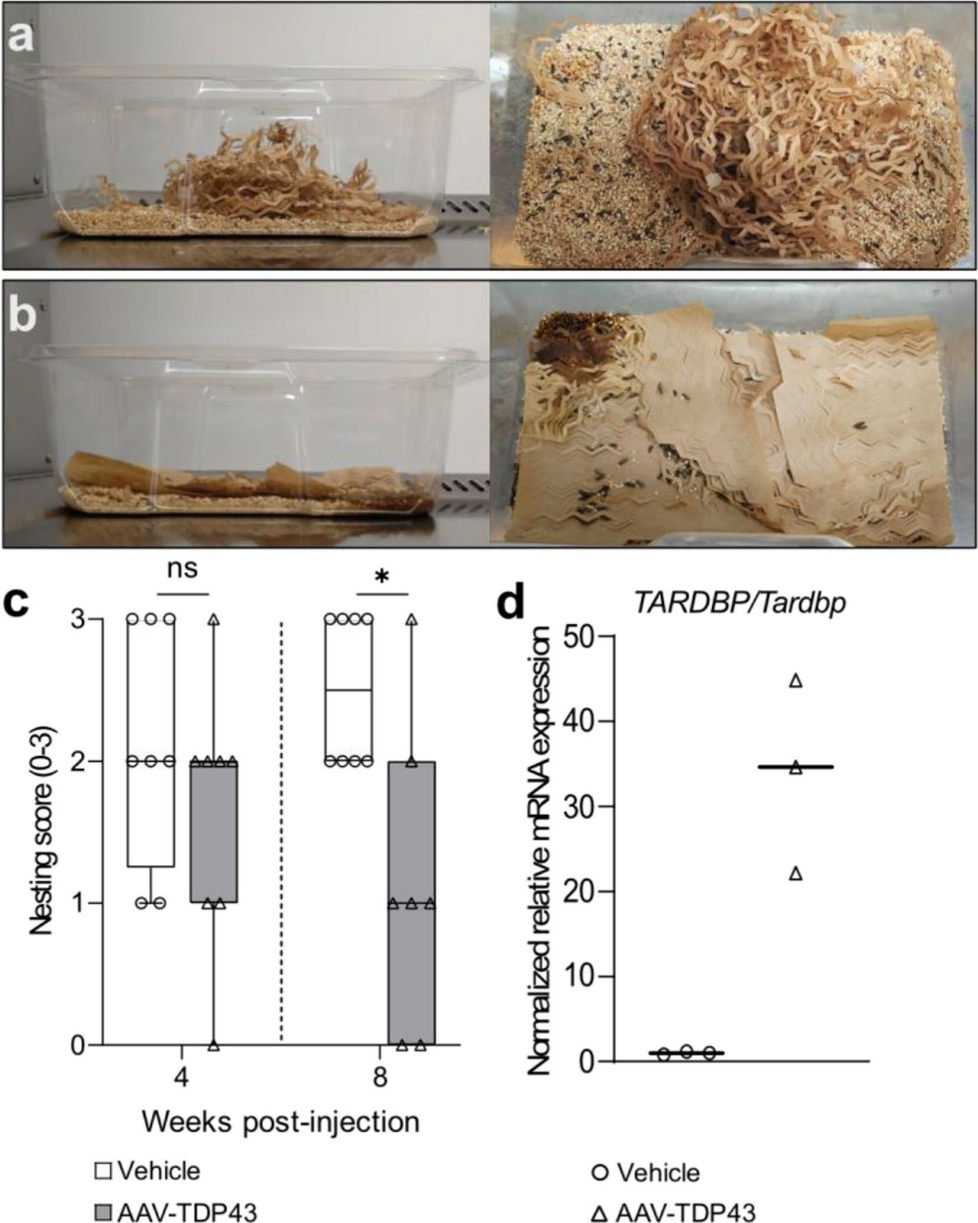
Mice overexpressing TDP-43 in the hypothalamus displayed reduced nesting behavior. Representative images of cages containing mice with hypothalamic injections of either vehicle control (**a**) or AAV-TDP43 (**b**), with the corresponding nesting scores are 3 (highest score) and 0 (lowest score), respectively. **c**. Mice overexpressing TDP-43 in the hypothalamus displayed decreased nesting behavior compared to control mice at 8 weeks post-injection (*p* = 0.016), but no difference was observed at 4 weeks post-injection. **d**. Increased levels of *TARDBP/Tardbp* mRNA confirmed TDP-43 overexpression in the hypothalamus. Data are presented as box-and-whisker plots with whiskers representing minimum and maximum values (**c**) or as median (**d**), * = *p* < 0.05 (2-tailed). Statistical analysis was performed using Mann-Whitney U test (**c**)

Mice overexpressing TDP-43 displayed reduced motor activity but preserved swimming ability, suggesting reduced motivation or drive, such as apathy, rather than severe motor impairment. Apathy is a common feature of patients with ALS/FTD [53, 70] as well as HD [19]. To investigate whether an apathy-like phenotype was present, another cohort of AAV-TDP43- and vehicle-injected mice was generated. Mice were housed in duplicate per treatment group for the analyses and nesting behavior (scored 0-3) was measured after 4 days at 4 and 8 weeks post-injection. No difference in nesting behavior was detected at 4 weeks post-injection (*p* = 0.290, *n* = 8 cages/group, Fig. 5a-c). However, mice overexpressing TDP-43 in the hypothalamus showed reduced nesting behaviour at 8 weeks post-injection compared to control mice injected with vehicle. The median nesting score of mice overexpressing TDP-43 was 1 (0-3, *p* = 0.016, *n* = 7 cages) compared to a median nesting score of 2 in vehicle-injected mice (2-3, *n* = 8 cages). These findings, combined with decreased motor activity, may suggest the development of apathy-like behavior in mice overexpressing TDP-43 in the hypothalamus.

In this cohort of mice, we confirmed overexpression of TDP-43 based on total *TARDBP/Tardbp* mRNA level using qRT-PCR (Fig. 5d). Median normalized relative mRNA levels were 34.6 (22.2-44.8, *n* = 3) in mice overexpressing TDP compared to 1.0 (0.8-1.2, *n* = 3) in vehicle-injected control mice. We also performed bulk RNA sequencing, which revealed alterations in genes associated with hypocretin/orexin, MCH, oxytocin, as well as neuropeptide Y and agouti-related peptide systems. Consistent with the neuropathological results in this study, mRNA levels of both the neuropeptide precursor and its corresponding receptor were reduced in the AAV-TDP43-injected mice (Table 1). Specifically, while the precursor for expression of MCH, oxytocin, and agouti-related peptide systems was significantly decreased, the hypocretin/orexin and neuropeptide Y precursors exhibited a trend towards decreased expression without achieving statistical significance. In contrast, the expression of all receptors was significantly downregulated in the AAV-TDP43-injected mice compared with the vehicle-injected control mice. This differential impact on precursor and receptor mRNA levels in the context of observed neuronal loss suggests a specific vulnerability or loss of cell populations in this model of TDP-43 overexpression.

**Table 1:** Overexpression of TDP-43 in the hypothalamus results in a specific decrease in neuropeptide-related transcripts

Neuropeptide pathway	Gene Symbol	Gene name	Log fold change	*P*-value
Hypocretin/Orexin	Hcrt	hypocretin	-0.59	0.0863
Hypocretin/Orexin	Hcrtr1	hypocretin receptor 1	-0.39	0.0206
Hypocretin/Orexin	Hcrtr2	hypocretin receptor 2	-0.74	< 0.0001
MCH	Pmch	pro-melanin-concentrating hormone	-0.49	0.0108
MCH	Mchr1	melanin-concentrating hormone receptor 1	-0.21	0.0947
Oxytocin	Oxt	oxytocin	-0.59	0.4389
Oxytocin	Oxtr	oxytocin receptor	-0.61	0.0093
Neuropeptide Y	Npy	neuropeptide Y	-0.15	0.6711
Neuropeptide Y	Npy1r	neuropeptide Y receptor Y1	-0.44	0.0079
Neuropeptide Y	Npy2r	neuropeptide Y receptor Y2	-0.46	0.1068
Neuropeptide Y	Npy5r	neuropeptide Y receptor Y5	-0.37	0.0414
Agouti-related peptide	Agrp	agouti related neuropeptide	-0.88	0.0990
Agouti-related peptide	Mc3r	melanocortin 3 receptor	-0.59	0.0022
Agouti-related peptide	Mc4r	melanocortin 4 receptor	-0.55	0.0444

### Nuclear and cytoplasmic localization of TDP-43 with inclusions following AAV-mediated TDP-43 expression in the hypothalamus

**Fig. 6:**
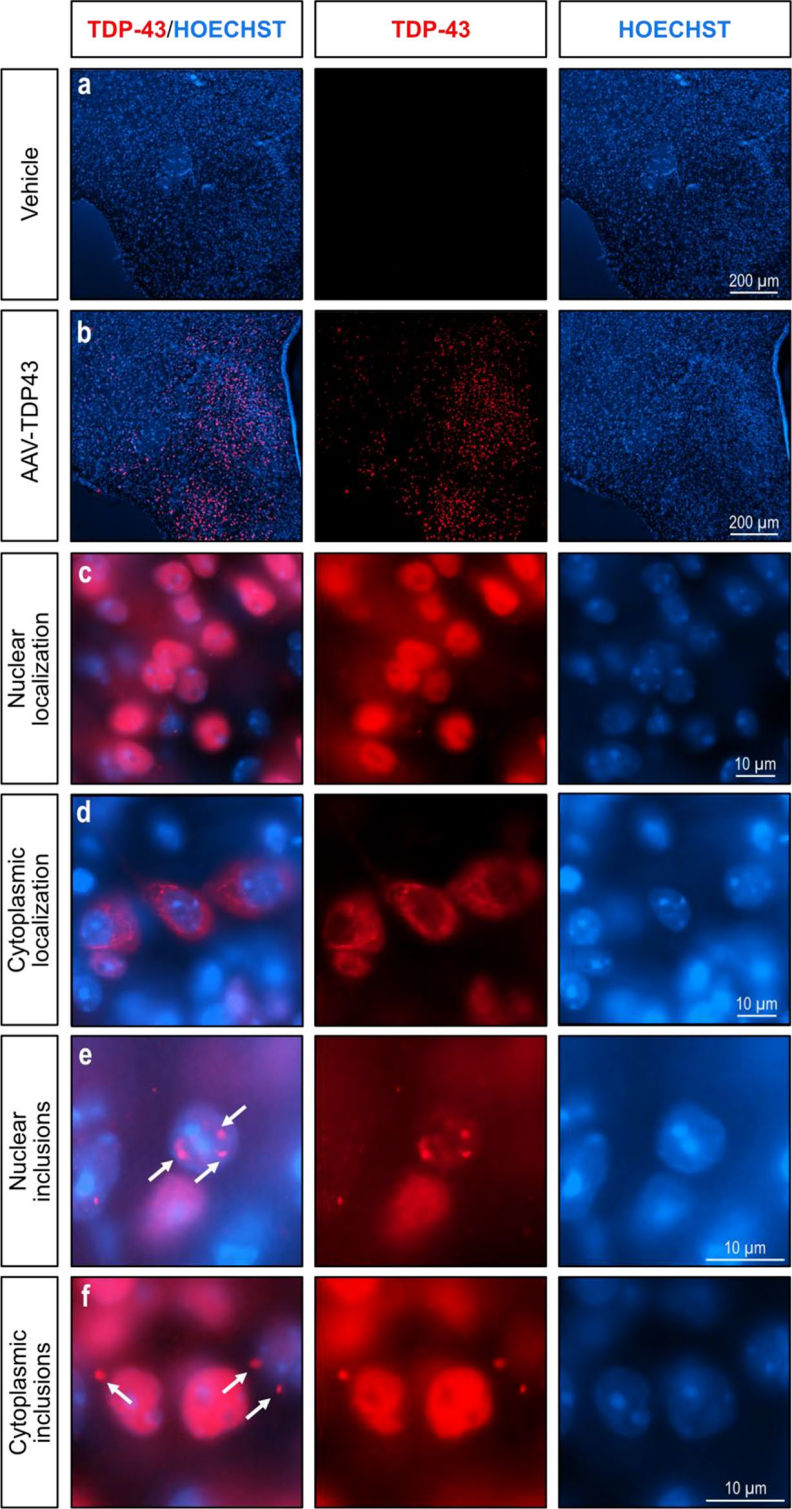
Nuclear and cytoplasmic localization of TDP-43 with inclusions following AAV-TDP43 vector injection in the hypothalamus of mice. Immunohistochemistry for TDP-43 was performed on brains with hypothalamic injections of either AAV-TDP43 (**a**) or vehicle (**b**) and counterstained with Hoechst for nuclear localisation. TDP-43 immunoreactivity was present in both the nucleus (**c**) and the cytoplasm (**d**). Both nuclear (**e**) and cytoplasmic (**f**) inclusions of TDP-43 were present

Pathogenic TDP-43 is known to undergo nuclear-to-cytoplasmic mislocalization and form both nuclear and cytoplasmic inclusions in patients with ALS/FTD [5, 37, 63]. To investigate the molecular effects following TDP-43 overexpression, we performed immunofluorescent analyses of brains from mice injected with AAV-TDP43 or vehicle in the hypothalamus 21 weeks post-injection. Brains were processed for immunofluorescence with an antibody against TDP-43 and counterstained with Hoechst for nuclear localization (Fig. 6a-b). TDP-43 was detected predominantly within the nucleus (Fig. 6c) but was also present in the cytoplasm (Fig. 6d). Furthermore, TDP-43-immunopositive inclusions were also present in both the nucleus (Fig. 6e) and cytoplasm (Fig. 6f), although they were more prevalent in the cytoplasm. Interestingly, we did not observe simultaneous overexpression of TDP-43 in both the nucleus and cytoplasm within the same cell. These results suggest that AAV-mediated TDP-43 overexpression in the hypothalamus recapitulates the molecular phenotype of patients with ALS/FTD, including TDP-43 mislocalization and the formation of both nuclear and cytoplasmic inclusions.

### Mice overexpressing TDP-43 in the hypothalamus exhibited significant repression of constitutive exons

**Fig. 7:**
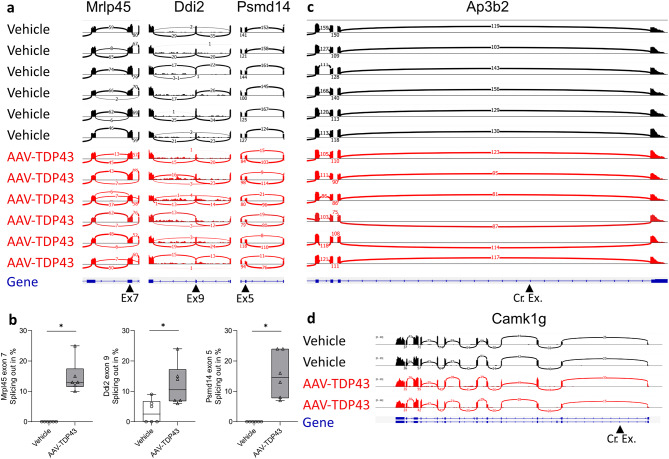
Overexpression of TDP-43 leads to skipping of specific constitutive exons, but not to the inclusion of neuron-specific cryptic exons. RNA-sequencing analysis of frozen hypothalamic tissues revealed several examples of exon skipping in Mrlp45, Ddi2, and Psmd14 (black arrowhead) in mice with TDP-43 overexpression (**a**). Quantification of events was performed by calculating the frequency of exon skipping events in the AAV-TDP43 or vehicle injected group (**b**). RNA-sequencing analysis does not reveal the expression of cryptic exons in Ap3b2 (**c**) Camk1g (**d**) (black arrowheads). Data are presented as box-and-whisker plots with whiskers representing minimum and maximum values, * = p < 0.05 (2-tailed). Stastical analysis was performed using Mann-Whitney U test (**b**)

To further determine the effects of TDP-43 overexpression on gene expression and splicing, we performed bulk RNA sequencing of enriched polyA RNA from the hypothalamus (Fig. 7a-b). Mice overexpressing TDP-43 displayed increased exon skipping on constitutive exons, such as Ddi2 exon 9 (2.5, 0-9%, *n* = 6 for vehicle; 10.5, 6-24%, *p* < 0.05, *n* = 6 for AAV-TDP43), Psmd14 exon 5 (0, 0%, *n* = 6 for vehicle; 14.5, 7-24%, *p* < 0.01, *n* = 6 for AAV-TDP43), and Mrld45 exon 7 (0, 0%, *n* = 6 for vehicle; 13, 10-25%, *p* < 0.01, *n* = 6 for AAV-TDP43), similar to what has been reported in the murine spinal cord overexpressing TDP-43 [15]. Analysis of the mapped RNA sequencing reads did not reveal expression of cryptic exons, including those previously described in Ap3b2 (Fig. 7c), and Camk1g (Fig. 7d) in mice after deletion of TDP-43 in excitatory neurons in cortex and hippocampus [44]. Visually inspection did not indicate obvious increases in cryptic exons in Stmn2, Sncb, and Unc13a (data not shown). These results indicate that TDP-43 overexpression in the hypothalamus results in significant repression of constitutive exons, without clear evidence of increased cryptic exons.

## Discussion

This study aimed to investigate whether there is a causative relationship between overexpression of wild-type human TDP-43 in the hypothalamus and development of neuropathology and metabolic and behavioral dysfunction. The wild-type form of TDP-43 is associated with the majority of familial and sporadic cases of ALS/FTD [54, 87] as well as to HD [7, 64, 73, 78]. We generated an AAV vector for targeted expression of TDP-43 in hypothalamic neurons of mice. Our results demonstrate a dose-dependent effect, where higher titers of the AAV-TDP43 vector were associated with cellular loss and the formation of ubiquitinated inclusions within the hypothalamus. Additionally, TDP-43 overexpression caused hypothalamic atrophy and a selective reduction in the number of neurons expressing hypocretin, MCH, and oxytocin. We also observed TDP-43 nuclear-to-cytoplasmic mislocalization and the formation of both intranuclear and cytoplasmic inclusions, which are hallmarks of ALS/FTD pathology [5, 37, 63]. Dysfunction of key metabolism and emotion regulation neuronal populations may drive the development of the metabolic changes and apathy-like phenotype we observed in mice overexpressing TDP-43 in the hypothalamus. The results from our novel AAV-TDP43 mouse model strengthens the growing body of evidence implicating wild-type TDP-43 in neurodegeneration [31, 56, 89, 90]. Our study also provides new insight on the hypothalamus as a critical region directly susceptible to TDP-43-mediated pathological effects and may contribute to early manifestation of clinical features observed in neurodegenerative disorders.

Selective loss of neuropeptide-expressing neuronal populations of the hypothalamus appears to be a common feature in several TDP-43 proteinopathies. We observed hypothalamic atrophy with loss of hypocretin-, MCH- and oxytocin-expressing neurons while sparing of vasopressin-expressing neurons in mice overexpressing TDP-43 consistent with previous studies in postmortem human ALS cases [12, 28]. Postmortem cases from persons with HD, Alzheimer’s disease and Parkinson’s disease also show loss of hypocretin-expressing neurons, but loss of oxytocin-expressing neurons has only been described in HD [6, 26, 29, 30, 52, 80, 88]. Loss of MCH neurons has also been reported in postmortem cases with Parkinson’s disease [80]. The degeneration of these neuropeptide-expressing neurons may play a role in the pathogenesis and development of the disease phenotype. For instance, near-complete loss of hypocretin neurons occurs in narcolepsy [81], and depletion of hypocretin in animal models leads to obesity and disruption of glucose homeostasis [27, 36, 43]. MCH deficiency has been linked to reduced food intake and weight loss in ALS patients [12, 49]. Furthermore, administration of MCH or a dual orexin receptor antagonist in ALS mice can ameliorate changes in sleep [35]. Oxytocin neuronal loss may at least partly explain social deficits seen in ALS/FTD and HD [9]. Further studies are however required to identify which specific neuronal populations in the hypothalamus are responsible for the link between TDP-43 overexpression and the development of metabolic and emotional dysregulation.

Metabolic dysregulation occurs in ALS, FTD and HD [22, 50]. In this study we show that overexpression of wild-type TDP-43 dysregulates metabolism, and leads to the development of obesity and hyperglycaemia without obvious effects on food intake. This result aligns with our previous studies where mutant huntingtin expression in the hypothalamus caused obesity and disrupted glucose homeostasis in mice [42, 75]. Metabolic assessment in metabolic cages using indirect calorimetry would be needed to further understand feeding behavior and energy expenditure after overexpression of TDP-43 in the hypothalamus. Importantly, the results on body weight gain are opposite to observations in persons with ALS and HD, who often exhibit reduced BMI even before the onset of motor symptoms [22, 50]. Potentially, it may be related to the increase in BMI reported in persons with FTD [3, 4], a disease with behavioral deficits more prominent than in ALS.

Our study shows that TDP-43 overexpression in the hypothalamus significantly reduces motor activity assessed by the open field, rotarod and elevated plus maze despite normal swimming ability. Importantly, the lateral hypothalamus is thought to provide an important drive to a number of vital behaviours including locomotion. Severe bilateral damage to the lateral hypothalamus is known to cause physical inactivity in mammals [51, 55]. Interestingly, recent studies have implicated several of the TDP-43 affected hypothalamic neuropeptide populations in regulating motor activity, including the hypocretin and the MCH systems [18, 46, 51, 79]. Our study also shows a deficit in nest-building behavior. This test is commonly used to assess motivation and daily activities in mice, indicative of goal-directed behavior and as a proxy to apathy [71]. Apathy, i.e. the lack of goal-directed behaviour and motivation is the most common behavioural change in the spectrum of ALS/FTD as well as other TDP-43 proteinopathies [1, 61, 77, 85]. The neurobiological substrate of apathy in neurodegenerative disorders has not been fully elucidated and there are no effective therapies to reduce apathy. The results from this study indicate that TDP-43 mediated pathology in the hypothalamus may be important in causing apathy-like behavior and further understanding of the underlying mechanisms may reveal novel targets for therapeutic interventions for apathy.

An important question is whether non-motor features manifest before motor and cognitive symptoms in ALS/FTD, and more broadly in TDP-43-proteinopathies. Interestingly, a recent study shows that sleep disturbances are present already in pre-symptomatic ALS risk gene carriers [35], which was also linked to loss of MCH neurons in the hypothalamus. Changes in BMI have also been detected in ALS risk gene carriers at pre-symptomatic stages, which were linked to reduced hypothalamic volume [34]. These results open for the possibility of the hypothalamus to be affected very early in the disease process. As hypothalamic neuronal populations are part of neurocircuitries to other brain regions, i.e. the cerebral cortex, the ventral striatum, the ventral tegmental area and the spinal cord, it is possible that pathological changes in the hypothalamus can exert direct effects also on these areas. Alternatively, pathology in these areas may also contribute to hypothalamic dysfunction. Further experimental studies using viral vector designs and conditional gene expression technologies may shed light on how different parts of the neurocircuitries may contribute to hypothalamic pathology and development of relevant phenotypes. This may also have relevance for the development of novel therapies as a recent study shows preservation of motor neurons in an animal model of ALS by treatment with the hypothalamic neuropeptide MCH [35].

TDP-43, which is natively a nuclear protein, is mislocalized to the cytoplasm and forms both nuclear and cytoplasmic inclusions in ALS/FTD cases [5, 37, 63]. Here, AAV-mediated TDP-43 overexpression resulted in TDP-43 localization to both the nucleus and the cytoplasm, with the formation of both nuclear and cytoplasmic inclusions. Hence, our model recapitulates the molecular phenotype of TDP-43 pathology. In a recent study, mis-localization of TDP-43 in zebrafish induced using a modified nuclear localization signal of endogenous Tardbp caused metabolic dysfunction with hypothalamic pathology [40], which aligns with the results of our study. Elevated nuclear TDP-43 and cytoplasmic mislocalization of TDP-43 could lead to both gain- and loss-of-function as TDP-43 plays an important role in the regulation of splicing [39]. High levels of nuclear TDP-43 have been proposed to results in constitutive exon skipping whereas low nuclear TDP-43 protein levels may lead to cryptic exon incorporation [15]. Here we show that hypothalamic TDP-43 overexpression leads to exon skipping similar to what has been reported previously in mouse models overexpressing TDP-43 in spinal cord [15]. Previous studies have shown that deletion of TDP-43 in excitatory neurons in the cerebral cortex and the hippocampus leads to generation of specific cryptic exons but we were not able to detect these in our model overexpressing TDP-43 [44]. This study also suggested that cryptic exons may be highly variable between cell types and may play a role in selective vulnerability to TDP-43. Hence, further studies on the generation of cryptic exons in specific hypothalamic neurons would be interesting.

The present study was designed to test the hypothesis that overexpression of wild-type TDP-43 could cause direct toxicity to hypothalamic neurons resulting in the development of changes in behavior and metabolism. Choices made in experimental designs come with different limitations. In this study, we chose to test our hypothesis in mice as we have previously established a model of hypothalamic expression of transgenes in this species using stereotactic delivery of AAV-vectors [42]. However, effects of disease-causing transgenes may be species-specific including TDP-43 mediated exon skipping [15] and similar studies in other species may therefore be interesting. Our AAV-vector based model uses the promoter synapsin to allow for neuronal expression and hence tests the effects of TDP-43 only in neurons. This choice was made as we have previously found inclusions of TDP-43 in several hypothalamic neuronal populations in postmortem cases with ALS [28]). It is not fully known how TDP-43 overexpression may affect glial cells in the hypothalamus. Delivery of AAV-vectors to specific brains regions could potentially lead to diffusion of the vector solution beyond the region of interest. For hypothalamic targeting, there is no promoter that can be used for pan-hypothalamic targeting. Further research could however employ promoters specific for certain neurocircuitries in the hypothalamus and/or use a flex-switch cre-recombinase model, similar to the one used in Bergh et al. to target mHTT to oxytocin neurons [11]. In the present study, we observed limited TDP-43 overexpression outside the hypothalamus with single cells overexpressing TDP-43 in the striatum, hippocampus and cortex. This could be due to TDP-43 propagation, which has previously been reported [45, 83], and requires further investigation. The use of appropriate controls to an AAV-vector mediated overexpression of a transgene is also important to consider. Here we used both AAV-vector mediated expression of GFP and another disease-causing protein, mHTT, as well as a vehicle solution. Although GFP did not show any neurotoxicity in this paradigm, other studies have demonstrated toxicity after AAV-mediated overexpression of GFP already at very low titers [48]. An empty AAV-vector could have been tested, although it does not control for overexpression of a protein and cannot be visualized in the targeted brain region. As sex differences may play a role in the sensitivity to disease causing genes and this study was conducted only in female mice, further analyses in male mice would be necessary for a broader generalization of the effects of TDP-43 in the hypothalamus. Moreover, interpretation of results from behavioral testing requires consideration of several parameters. Effects on activity including reduced nesting behavior could suggest apathy-like behavior but would be interesting to explore further using other tests assessing motivation. The concurrent manifestation of both a metabolic phenotype with obesity and a behavioral phenotype with reduced motor activity also complicates the interpretation of the data. Obesity could result in reduced motor activity and reduced activity could contribute to weight gain. Further research is therefore needed to understand the temporal relationships between these different phenotypes.

**Fig. 8:**
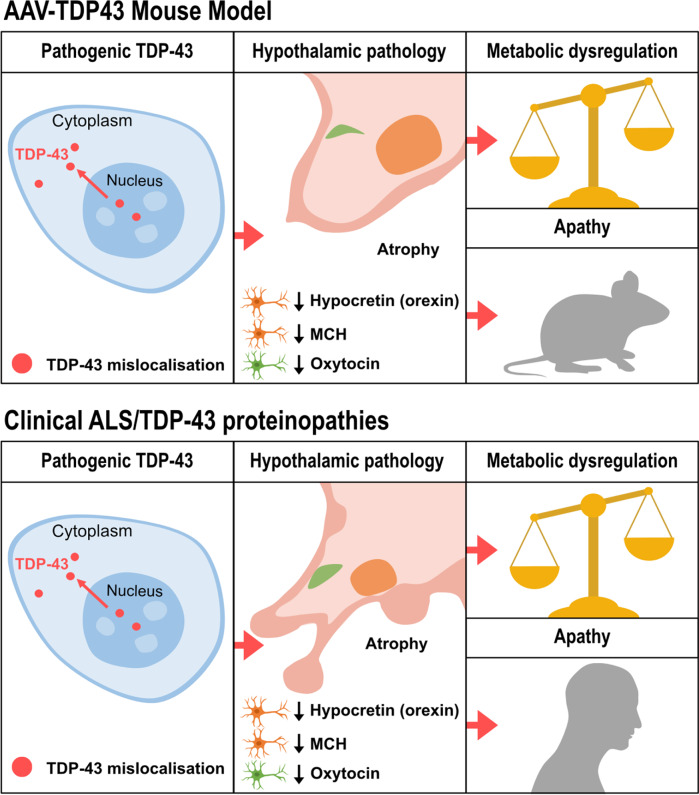
Schematic representation of the molecular phenotype, hypothalamic pathology, and behavioral dysfunction induced by AAV-mediated overexpression of human wild-type TDP-43 in the hypothalamus, recapitulating key neuropathological features observed in clinical ALS and metabolic and psychiatric features seen in TDP-43 proteinopathies

In conclusion, this study shows that wild-type TDP-43 overexpression in the hypothalamus can lead to the development of neuropathological and molecular features similar to what have been reported for ALS/FTD including hypothalamic pathology with TDP-43 mislocalization and formation of TDP-43 nuclear and cytoplasmic inclusions (Fig. 8). TDP-43 overexpression in the hypothalamus triggers a cascade of pathological events leading to metabolic and emotional alterations, likely involving dysfunction and/or loss of neuronal population expressing hypocretin, MCH and oxytocin. These results suggest that wild-type human TDP-43 can exert gain-of-toxic function within the hypothalamus, with the potential to contribute to the pathogenesis of neurodegenerative diseases. Therefore, elucidating the molecular and cellular mechanisms of the effects of TDP-43 in the hypothalamus may provide novel therapeutic targets for TDP-43 proteinopathies.

## Electronic supplementary material

Below is the link to the electronic supplementary material.

Supplementary Material 1

## Data Availability

The datasets analysed during the current study are available from the corresponding author on reasonable request.

## Abbreviations

AAV: Adeno-associated virus
AAV-GFP: AAV-vector expressing GFP
AAV-mHTT: AAV-vector expressing huntingtin with 78 polyglutamines
AAV-TDP43: AAV-vector expressing TDP-43
ALS: Amyotrophic lateral sclerosis
BMI: Body mass index
cpm: Counts per million
CV: Cresyl violet
DAB: 3,3’-diaminobenzidine
DEXA: Dual energy X-ray absorptiometry
FTD: Frontotemporal dementia
gc: Genome copies
GFP: Green fluorescent protein
GFAP: Glial neurofibrillary protein
HD: Huntington disease
Iba1: Ionized calcium binding adaptor molecule 1
KPBS-T: Triton X-100 in potassium phosphate-buffered saline
MCH: Melanin-concentrating hormone
mHTT: Mutant huntingtin
PBS: Phosphate-buffered saline
PFA: Paraformaldehyde
RIN: RNA integrity number
rpm: Revolutions per minute
TDP-43: TAR DNA-binding protein 43
