# Supplementary material for: TDP-43 overexpression in the hypothalamus drives neuropathology, dysregulates metabolism and impairs behavior in mice
Source: Acta Neuropathol Commun. 2025 May 27;13:119. doi: 10.1186/s40478-025-02018-8 (PMC12108026; doi:10.1186/s40478-025-02018-8)
Supplement: Supplementary file 1 — Supplementary Material 1 [file 40478_2025_2018_MOESM1_ESM.docx]

**Supplementary information:**

**TDP-43 overexpression in the hypothalamus drives neuropathology, dysregulates metabolism and impairs behavior in mice**

Sofia Bergh^1^, Nicolas Casadei^2,3^, Sanaz Gabery^1^, Oskar Simonsson^1^, João M.N. Duarte^4,5^, Deniz Kirik^6^, Huu Phuc Nguyen^7^ and Åsa Petersén^1,8^

^1^Translational Neuroendocrine Research Unit (TNU), Department of Experimental Medical Science, Lund University, Lund, Sweden

^2^Institute of Medical Genetics and Applied Genomics, University of Tübingen, Tübingen, Germany

^3^NGS Competence Center Tübingen, Tübingen, Germany

^4^Diabetes and Brain Function Unit, Department of Experimental Medical Science, Lund University, Lund, Sweden

^5^Wallenberg Centre for Molecular Medicine, Lund University, Lund, Sweden

^6^Brain Repair and Imaging in Neural Systems (BRAINS), Department of Experimental Medical Science, Lund University, Lund, Sweden

^7^Department of Human Genetics, Medical Faculty, Ruhr University Bochum, Bochum, Germany

^8^Department of Psychiatry, Skåne University Hospital, Lund, Sweden

## **Corresponding author:**

Correspondence should be addressed to Åsa Petersén (email:asa.petersen@med.lu.se), Translational Neuroendocrine Research Unit, Department of Experimental Medical Science, Lund University, BMC D11, 221 84 Lund, Sweden.

**Supplementary Figure 1**


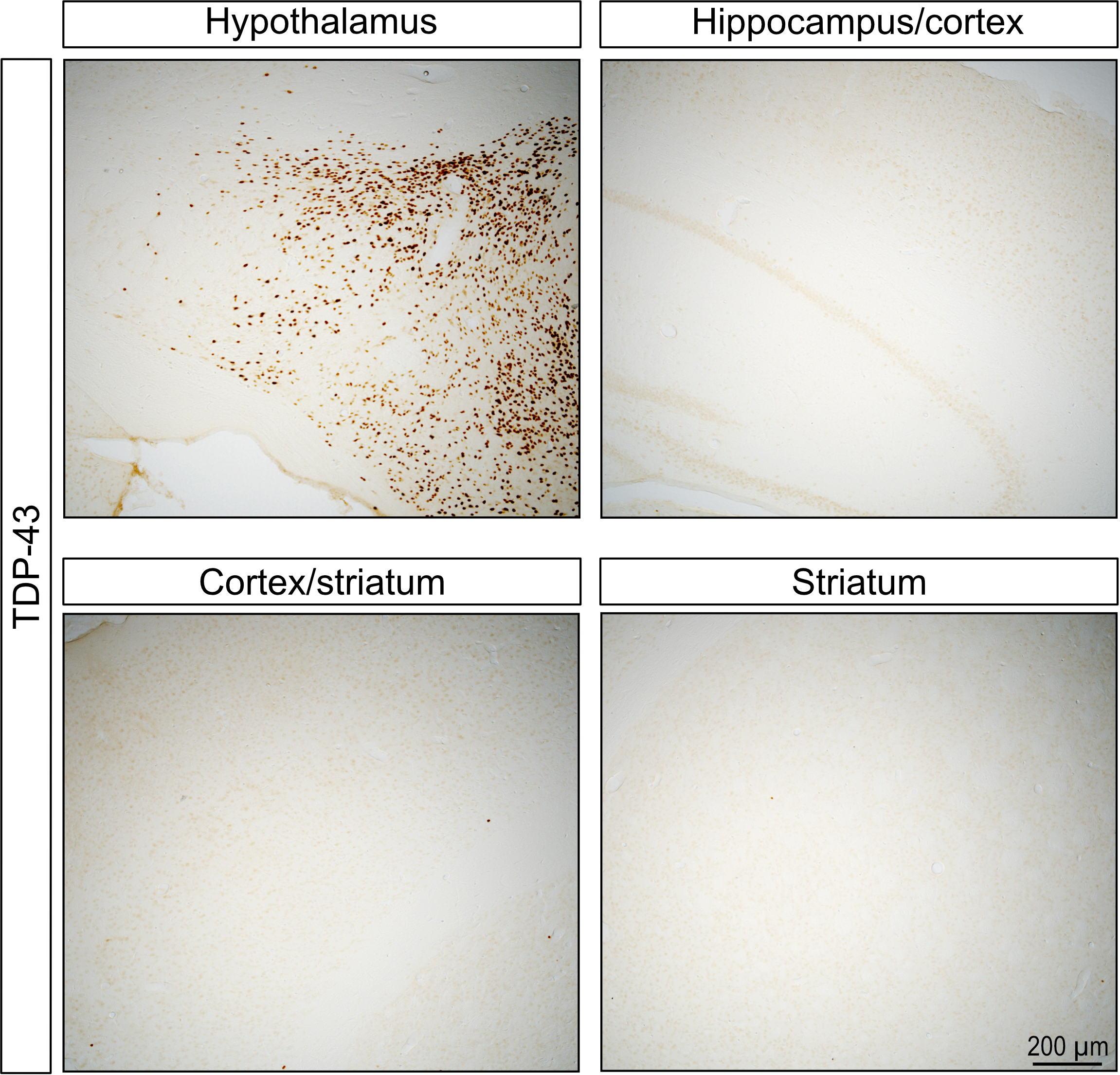


**Supplementary Figure 1**. Mice injected with the AAV-TDP43 vector that expresses TDP-43 under the neuron-specific synapsin-1 promoter exhibited TDP-43 immunoreactivity primarily in the hypothalamus, with single TDP-43 overexpressing cells observed in the cerebral cortex, striatum and hippocampus.

**Supplementary Figure 2**


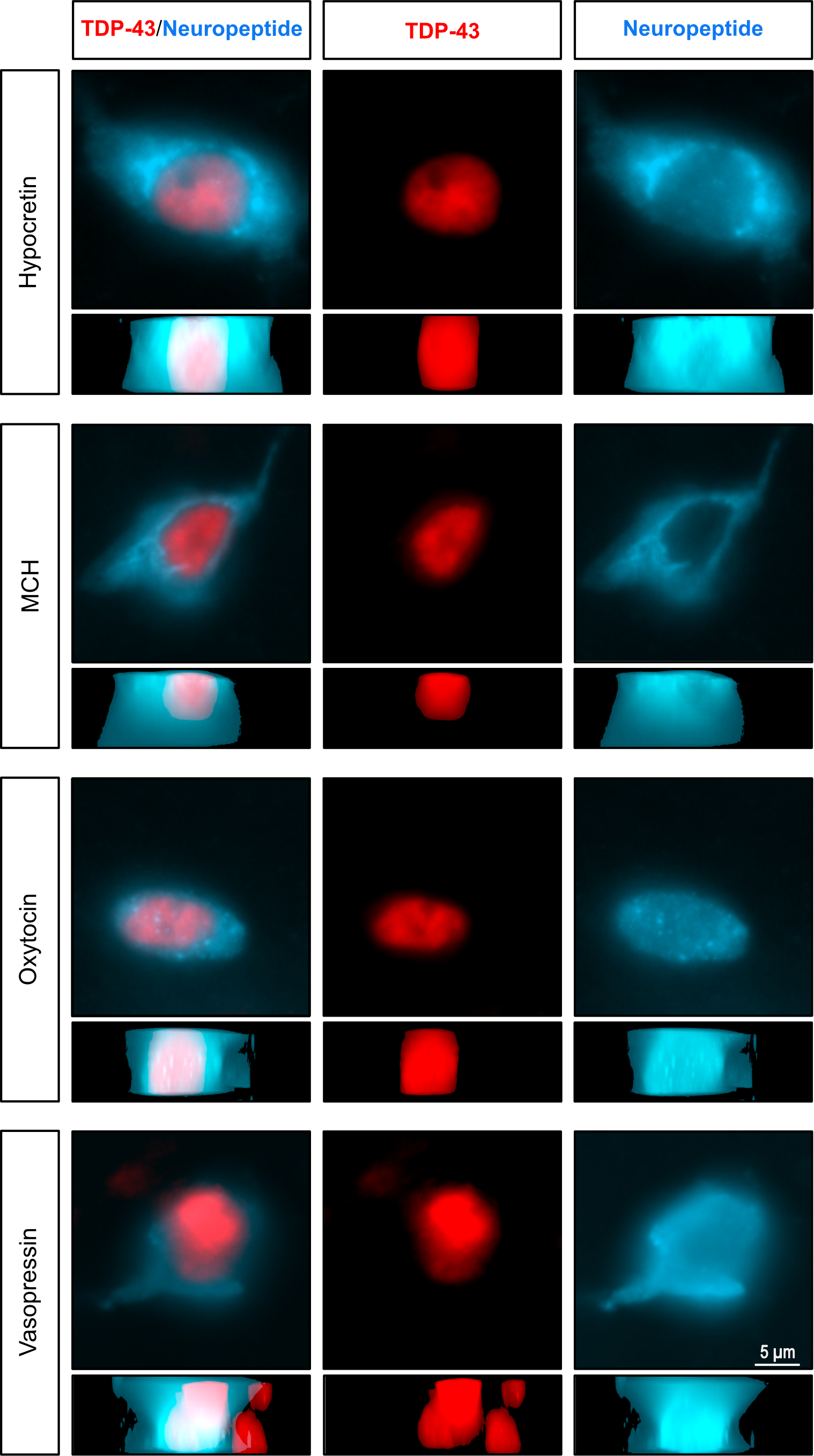


**Supplementary Figure 2.** Targeting of hypothalamic neuropeptide-expressing neurons by AAV-TDP43. TDP-43 overexpression was confirmed in hypocretin (orexin), melanin-concentrating hormone (MCH) oxytocin and vasopressin with representative z-stacks showing co-localization of TDP-43 and the neuropeptide-expressing neurons.

**Supplementary Figure 3**

**
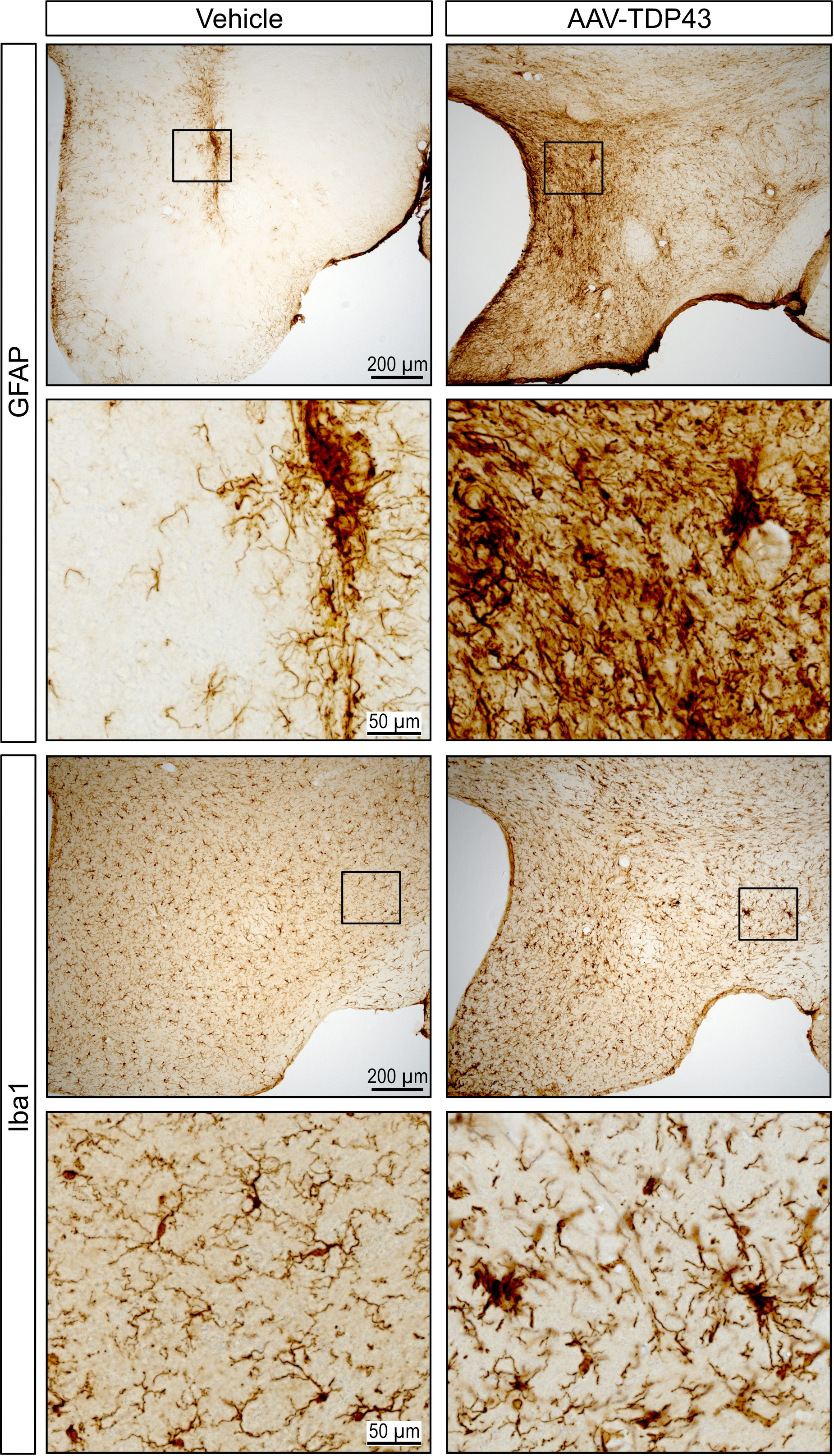
**

**Supplementary Figure 3**. Immunohistochemical analyses of glial neurofibrillary protein (GFAP) and ionized calcium binding adaptor molecule 1 (Iba1) revealed increased GFAP-immunoreactivity and altered Iba1-immureactive cell morphology (hyperramification with thickening of processes), indicative of astrogliosis and microglia activation in TDP-43 overexpressing mice compared to vehicle-injected controls. Vehicle-injected controls showed increased GFAP immunoreactivity surrounding the stereotaxic injection needle track.

**Supplementary Figure 4**


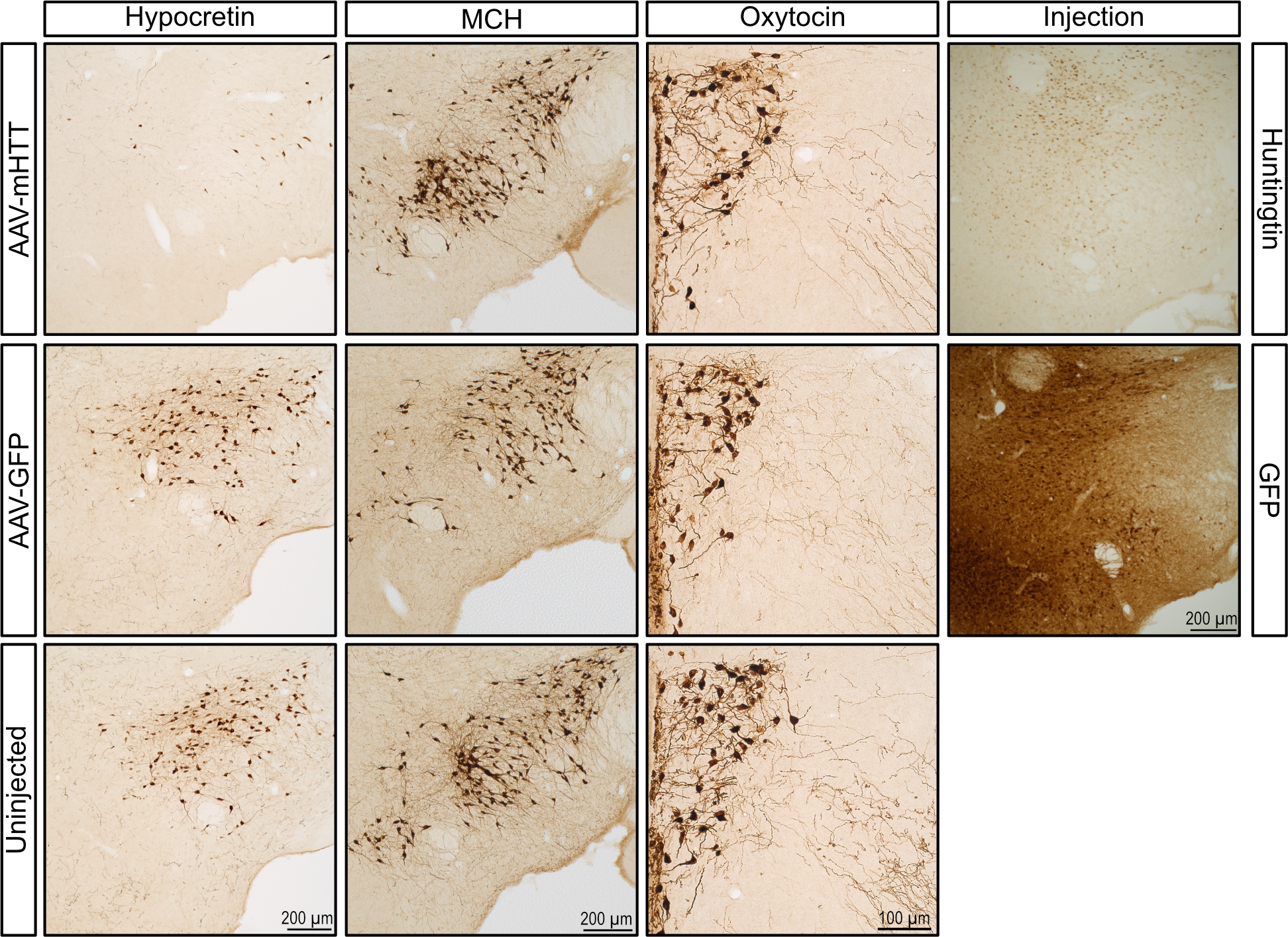


**Supplementary figure 4**. At 10 weeks post-injection, mice overexpressing mutant huntingtin (AAV-mHTT) appeared to have a recued number of hypocretin-expressing neurons, in line with what we have reported previously [42], while mice overexpressing GFP (AAV-GFP) showed no loss of hypocretin-, melanin concentrating hormone (MCH)-, or oxytocin-expressing neurons.
